# A novel prognostic risk model for patients with refractory/relapsed acute myeloid leukemia receiving venetoclax plus hypomethylating agents

**DOI:** 10.1038/s41375-024-02501-6

**Published:** 2025-01-08

**Authors:** Rabia Shahswar, Razif Gabdoulline, Katja Krueger, Martin Wichmann, Katharina S. Götze, Krischan Braitsch, Manja Meggendorfer, Laura Schmalbrock, Lars Bullinger, Franziska Modemann, Walter Fiedler, Juergen Krauter, Stephan Kaun, Susanne Rotermund, Andreas Voß, Yvonne Lisa Behrens, Anke Katharina Bergmann, Elisabeth Koller, Gernot Beutel, Felicitas Thol, Florian Heidel, Michael Heuser

**Affiliations:** 1 Department of Hematology, Hemostasis, Oncology, and Stem Cell Transplantation, Hannover Medical School, Hannover, Germany; 2 Department of Medicine III, Technical University of Munich (TUM) School of Medicine and Health, Munich, Germany; 3 Bavarian Center for Cancer Research (BZKF), Erlangen, Germany; 4 MLL, Munich Leukemia Laboratory, Munich, Germany; 5 Department of Hematology, Oncology, and Tumor Immunology, Charité-Universitätsmedizin Berlin, Berlin, Germany; 6 Department of Oncology, Hematology and Bone Marrow Transplantation, University Cancer Center Hamburg, University Medical Center Hamburg-Eppendorf, Hamburg, Germany; 7 Department of Internal Medicine, Municipal Hospital Braunschweig, Braunschweig, Germany; 8 Department of Internal Medicine, Municipal Hospital Bremen-Mitte, Bremen, Germany; 9 Department of Oncology and Hematology, University Clinic Oldenburg, Oldenburg, Germany; 10 Department of Human Genetics, Hannover Medical School, Hannover, Germany; 11 3rd Medical Department, Hanusch Hospital, Vienna, Austria; 12 Department of Internal Medicine IV, University Hospital Halle (Saale), Martin-Luther-University Halle-Wittenberg, Halle, Germany

**Keywords:** Acute myeloid leukaemia, Cancer genetics

## Abstract

Off-label hypomethylating agents and venetoclax (HMA/VEN) are often used for relapsed and refractory (R/R) AML patients. However, predictors of outcome are elusive. The objective of the current retrospective observational multicenter study of 240 adult patients (median age 68.6 years) with R/R AML was to establish a prognostic risk score. Overall response was documented in 106 (44%) patients. With a median follow-up of 31.5 months, 179 deaths were recorded. Median overall survival (mOS) was 7.9 months. In multivariate analysis of the subgroup with molecular information (*n* = 174), risk factors for inferior survival included the presence of extramedullary disease, HMA pretreatment and mutations in *NF1*, *PTPN11*, *FLT3*, and *TP53*, whereas mutated *SF3B1* was identified as favorable risk factor. These risk factors were subsequently applied to construct an HR-weighted risk model that allocated patients to one of three risk groups with significantly different survival outcomes: favorable (*n* = 46; mOS 21.4 months), intermediate (*n* = 75; mOS 7.5 months), and adverse (*n* = 53; mOS 4.6 months; *p* < 0.001). The model was validated in 189 AML patients treated with HMA/VEN in first line. This clinical-molecular, 3-tiered **ven**etoclax **p**rognostic **r**isk **s**core (VEN-PRS) for HMA/VEN treatment outcomes in R/R AML patients will support the selection of appropriate treatment options in this high-risk population.

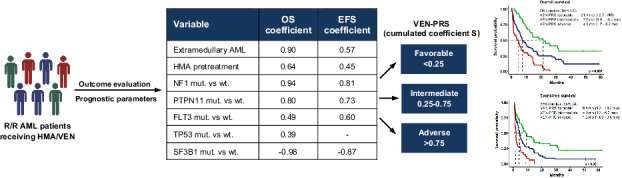

## Introduction

Despite substantial progress in the development of new therapeutic strategies, relapsed and refractory (R/R) acute myeloid leukemia (AML) is associated with poor outcome and still represents an underserved medical condition [1]. The prognosis of R/R AML patients depends on many factors including age, prior therapy including allogeneic hematopoietic cell transplantation (alloHCT), timing of relapse, and the mutational as well as the cytogenetic profile of the disease at relapse [2]. According to the European LeukemiaNet (ELN) primary refractory disease is defined as failing to achieve complete remission (CR) after two cycles of intensive chemotherapy (including at least one cycle of intermediate dose cytarabine) [3]. Ferguson et al. additionally defined an insufficient response to the first induction – i.e. less than 50% reduction in blasts and the presence of more than 15% blasts – as primary refractory disease since it was shown that these patients have an equally poor prognosis as patients with primary refractory disease defined by ELN [4]. Primary refractory disease is reported in approximately 25–30% of patients and is associated with poor outcome due to limited salvage options with low response rates [5]. A retrospective study by Zeichner et al. reported a median overall survival (OS) of only 4 months in patients with R/R AML [6]. Risk factors associated with primary refractory AML include a complex or monosomal karyotype, advanced age, and an increased time to blast clearance in the bone marrow or peripheral blood [7–9]. In patients with hypomethylating agents, the current ESMO Clinical Practice Guideline suggests to assess response at the very least after four cycles of non-intensive therapy to diagnose refractory disease [10].

Relapse is diagnosed in patients who have achieved a CR or CR with incomplete hematologic recovery (CRi) but show a reappearance of blasts in the bone marrow ≥5%, reappearance of blasts in the blood in at least 2 peripheral blood samples at least one week apart, or the development of extramedullary AML [3]. In a large analysis of more than 3000 patients treated with intensive frontline chemotherapy, approximately 60% of patients suffered from relapse after a median of 7.2 months after achieving a CR [11].

In patients with relapsed AML treated with intensive chemotherapy, Breems et al. identified patient and disease characteristics to predict outcomes of patients in first relapse. Based on the parameters length of relapse-free interval after first CR, cytogenetics at diagnosis, age at relapse, and prior alloHCT, patients were stratified into favorable-, intermediate-, or poor-risk groups, with associated 5-year OS of 46%; 18% and 4%, respectively [12].

There is no standard salvage therapy in R/R AML, particularly in older patients [3, 10]. Feasible options include enrollment into clinical trials and use of targeted therapies such as gilteritinib for AML with *FLT3* mutation [10, 13], combination of the BCL-2 inhibitor venetoclax (VEN) with HMA, or cytarabine-based salvage chemotherapy [14, 15]. Response to HMA/VEN salvage treatment is reported in 20–40% of R/R AML patients with a median OS of 3.3–11.2 months [16–21].

However, prognostic factors associated with outcome to venetoclax-based regimens remain sparse. Therefore, precise, robust and applicable prognostic models for predicting long-term survival, stratifying risk groups, and helping with therapeutic decision-making in R/R AML is still an unmet clinical need.

In the current study of 240 patients with R/R AML receiving HMA and VEN we aimed to (I) assess efficacy of HMA/VEN in a large cohort of R/R AML patients, (II) determine clinical and genetic predictors of response, event-free survival (EFS) and OS, and (III) develop an integrative response, EFS and OS model to support treatment decisions.

## Patients and methods

Patients aged 18 years or older with refractory or relapsed (R/R) acute myeloid leukemia (AML) as defined by the 2022 International Consensus Classification of Myeloid Neoplasms and Acute Leukemias (ICC) [22], who had been treated with VEN combined with HMA and had been reported to the venetoclax registry (venreg.org; ClinicalTrials.gov NCT03662724) were included in the analysis. The venetoclax registry is a multicenter, prospective, observational cohort study initiated in 2018, which allowed retrospective patient inclusion until 2017 [23]. In the current analysis patients were included from eight academic centers in Germany and Austria. Patients receiving low-dose cytarabine or other non-HMA combinations with VEN were excluded. Extramedullary disease was evaluated clinically and considered as reported by the investigators to the registry. All patients provided written informed consent to the off-label use of VEN, genetic analysis and use of clinical data according to the Declaration of Helsinki and institutional guidelines. Response and outcome data are reported for the entire cohort. The subgroup of patients with available molecular data (*n* = 174) constitute the molecular cohort for risk model construction. A cohort of 189 AML patients receiving HMA/VEN as first line treatment from the same registry served as an independent validation cohort. Details of the first line cohort will be reported separately. The registry was approved by the local Ethics Review Committee (ethical vote No.7972_BO_K_2018).

### Treatment

All patients had received off-label VEN for relapsed or refractory disease in combination with either azacitidine 75 mg/m^2^ days 1–7 or 1–5 and 8 + 9 subcutaneously (*n* = 192) or decitabine 20 mg/m^2^ days 1–5 intravenously (*n* = 48). Patients had not received VEN in a prior line of therapy. Orally administered VEN doses ranged from 50 to 600 mg depending on concomitant CYP3A4 inhibitor use for antifungal prophylaxis. Additionally, patients received supportive care measures including transfusions, acid-reducing and antiemetic agents according to local practice. Timing of response evaluation was at the discretion of the treating physician, and bone marrow was obtained for response assessment after the completion of either one or two cycles in the majority of patients.

Details of cytogenetic, molecular and statistical analyses can be found in the Supplemental Materials.

### Comprehensive risk model

Based on multivariate Cox regression a model was derived capable of predicting personalized outcomes. The OS prediction is performed by using ***N*** patient-dependent parameters ${{{\bf{X}}}}=({X}_{1},{X}_{2},\ldots ,\,{X}_{N})$ of the multivariate model in the Cox proportional hazards formula:

$$h\left(t,{{{\bf{X}}}}\right)={h}_{0}\left(t\right)\cdot \exp ({{\sum}_{{{{\rm{i}}}}}{{{\rm{\beta }}}}}_{{{{\rm{i}}}}}\cdot {X}_{i})$$

where ${h}_{0}\left(t\right)$ is a baseline hazard, a hazard for a patient with zero in every variable and *β*_*i*_ are coefficients of the model for the parameter *X*_*i*_, related to the respective hazard ratio as ${h}_{i}=\exp ({\beta }_{{{{\rm{i}}}}})$. The same formula was used for EFS with its own coefficients. The value of the sum $S={{\sum }_{{{{\rm{i}}}}}\beta }_{{{{\rm{i}}}}}\cdot {X}_{{i}}$ is defined as venetoclax-prognostic risk score (VEN-PRS) for each patient. For OS/EFS the score value S_OS_/_EFS_ < 0.25 corresponds to a favorable risk, 0.25 < S_OS_/_EFS_ ≤ 0.75 to intermediate risk, and S_OS_/_EFS_ > 0.75 to adverse risk. Further details are provided in the Supplemental Materials.

To evaluate calibration of the model, the patient population was divided into a training cohort comprising 100 patients and a validation cohort comprising 74 patients. Two hundred models were generated by randomly choosing 100 training patients and 74 test patients and the predicted and observed 10-month OS and EFS times were compared for three risk groups. The discrimination ability of the model was evaluated by area under the curve (AUC) values of receiver operating characteristic (ROC) curves for 10-month OS and EFS [24].

All statistical analyses were performed using the R computing language (version 4.2.2) and statistical software package SPSS 29.0.

## Results

### Patient characteristics

Two-hundred-and-forty R/R AML patients were treated with HMA and off-label VEN between January 2017 and April 2023 and were included in our analysis (entire cohort). Table 1 provides details on clinical characteristics at the time of treatment with HMA/VEN. The median age was 68.6 years (range, 20-88). A similar proportion of patients had relapsed (*n* = 110; 46%) or had refractory disease (*n* = 130; 54%). ELN 2022 risk was favorable, intermediate and adverse in 5%, 20%, and 57% of patients, respectively. Patients had a median of two prior treatment lines (range, 1–7), including 67% patients with intensive pretreatment and 36% with prior alloHCT. The majority of patients received azacitidine (80%) combined with VEN, while 20% received decitabine (Table 2). Median VEN dose after ramp-up was 100 mg (range, 50–600 mg) and 121 patients (50%) received VEN for 14 days.

**Table 1:** Demographic and disease characteristics of patients with relapsed/refractory AML treated with HMA/VEN.

Patient and disease characteristics	Entire cohort (*N* = 240)	Molecular cohort (*n* = 174)
Age		
Median (years, range)	67 (20–88)	69 (24–88)
Sex, *n* (%)		
Male	138 (58)	98 (56)
Female	102 (42)	76 (44)
ECOG, *n* (%)		
0	54 (29)	35 (24)
1	98 (52)	80 (54)
2	27 (14)	24 (16)
3 and 4	10 (5)	8 (5)
Missing	51	27
ICC 2022 classification, *n* (%)		
AML with recurrent genetic abnormality	18 (8)	16 (9)
AML with MRGM	61 (25)	57 (33)
AML with MRCA	72 (30)	56 (32)
AML with mutated *TP53*	11 (5)	9 (5)
AML not otherwise specified	78 (32)	36 (21)
ICC 2022 diagnostic qualifiers, *n* (%)		
De novo	124 (52)	83 (48)
Therapy-related	12 (5)	9 (5)
Progressed from MDS	84 (35)	64 (37)
Progressed from MDS/MPN	19 (8)	17 (10)
Germline predisposition	–	–
Missing	1	1
ELN 2022 risk group, *n* (%)		
Favorable	12 (6)	9 (6)
Intermediate	47 (24)	39 (25)
Adverse	136 (70)	108 (69)
Missing	45	18
Complex karyotype, *n* (%)		
Yes	70 (34)	55 (36)
No	135 (66)	98 (64)
Missing	35	21
Extramedullary disease, *n* (%)		
Yes	20 (8)	17 (10)
No	218 (92)	156 (90)
Missing	2	1
Relapsed/refractory disease, *n* (%)		
Relapsed	110 (46)	79 (45)
Refractory	130 (54)	95 (55)
Time to relapse before VEN, *n* (%)	*n* = 110	*n* = 79
≤ 6 months	21 (36)	19 (37)
7–18 months	23 (39)	23 (44)
>18 months	15 (25)	10 (19)
Missing	51	27
Median time from CR to relapse before VEN, months (range)	*n* = 110 11.3 (1.7–109.6)	*n* = 79 8.6 (1.7–76.0)
WBC count at start of VEN (×10^9^/L)		
Median (range)	2.69 (0.3–224.6)	3.17 (0.1–76)
Hemoglobin at start of VEN (g/dL)		
Median (range)	9.0 (6.3–14.4)	9.0 (6.3–14.4)
Platelet count at start of VEN (×10^9^/L)		
Median (range)	34 (1–742)	33 (1–496)
Blasts in BM at start of VEN (%)		
Median (range)	40 (6–99)	30 (6–99)
Blasts in PB at start of VEN (%)		
Median (range)	5.8 (0–96)	5 (0–96)
Functional categories of mutated genes, *n*		
Cohesin complex	29 (12)	29 (17)
Epigenetic modifiers	108 (45)	108 (62)
Nucleophosmin	19 (8)	19 (11)
Signal transduction	84 (35)	84 (48)
Spliceosome	51 (21)	51 (29)
Transcription factor	95 (40)	95 (55)
Tumor suppressor	45 (19)	45 (26)
No mutation	19 (8)	19 (11)
Prior lines of treatment, *n*		
Median (range)	2 (1–7)	2 (1–7)
Prior alloHCT, *n* (%)		
Yes	83 (36)	61 (37)
No	148 (64)	104 (63)
Missing	9	9
Prior intensive chemotherapy, *n* (%)		
Yes	162 (67)	114 (66)
No	78 (33)	60 (34)
Prior HMA treatment, *n* (%)	132 (55)	91 (52)

*alloHCT* allogeneic hematopoietic cell transplantation, *BM* bone marrow, *ELN* European LeukemiaNet, *ICC* International Consensus Classification, *HMA* hypomethylating agents (i.e. azacitidine, decitabine), *MRCA* myelodysplasia-related cytogenetic abnormality, *MRGM* myelodysplasia-related gene mutation, *p* *P*-value, *PB* peripheral blood, *VEN* venetoclax, *WBC* white blood count.

**Table 2:** Treatment characteristics of patients with relapsed/refractory AML treated with HMA/VEN (entire cohort).

Parameter	HMA/VEN R/R (*N* = 240)
Combination partner, *n* (%)	
Azacitidine	192 (80)
Decitabine	48 (20)
VEN steady state dose, n (%)	
50/70 mg	9 (4)
100 mg	149 (63)
400 mg	69 (29)
Miscellaneous doses	10 (4)
Missing	3
Median (range)	100 (50–600)
Inpatient/Outpatient at start VEN, *n* (%)	
Inpatient	164 (72)
Outpatient	63 (28)
Missing	13
Dose ramp-up, *n* (%)	
Yes	81 (36)
No	146 (64)
Missing	13
VEN duration per cycle, *n* (%)	
28 days	91 (39)
14 days	121 (52)
Others	19 (8)
Missing	9
Duration of VEN treatment, months	
Median (range)	1.78 (0.1–33.93)
Concomitant use of azoles, *n* (%)	
Yes	162 (78)
No	47 (22)
Missing	31
Number of treatment cycles	*n* = 231
Median (range)	2 (1–34)
Use of G-CSF, *n* (%)	
Yes	38 (17)
No	192 (83)
Missing	10
Subsequent therapies after HMA/VEN treatment	
Yes	113 (54)
Chemotherapy	38 (18)
AlloHCT	49 (23)
DLI	19 (9)
BSC	7 (3)
Died/no further treatment	98 (46)
Missing	29
Time from HMA/VEN start to subsequent treatment, months	
Median (range)	0.83 (0.1–8.0)

*alloHCT* allogeneic hematopoietic cell transplantation, *BSC* best supportive care, *G-CSF* granulocyte colony-stimulating factor, *HMA* hypomethylating agents (i.e. azacitidine, decitabine), *VEN* venetoclax.

For 174 patients (73%) molecular data were available (molecular cohort, Supplementary Fig. S1).

Most commonly mutated genes included those associated with clonal hematopoiesis (*TET2* 20%, *ASXL1* 18%, *DNMT3A* 17%) besides *RUNX1* (19%), and *TP53* (18%), whereas 19 patients (11%) had no detectable mutation. Among those, three patients had a complex karyotype, three had *KMT2A*-rearranged AML and two had single cytogenetic aberrations, while the remaining 11 (6%) patients had no detectable cytogenetic aberration. Median variant allele frequencies (VAF) are depicted in Supplementary Fig. S2, indicating lower VAFs in genes that are involved in signaling pathways such as *FLT3-*ITD, *NRAS* and *PTPN11*, whereas higher VAFs were observed in splicing genes (*SRSF2, ZRSR2*) and transcription factors (*RUNX1*).

### Treatment response and outcome in the entire cohort

Patients received a median of 2 treatment cycles (range 1-34) of HMA/VEN. CR and CRi rates were 25% and 14%, respectively. The overall response rate (ORR) was 44%, including 39% of patients with CR/CRi and 5% of patients with MLFS (Table 3). Partial remission was achieved in one patient, while 45% of patients (*n* = 108) did not respond to HMA/VEN. Twenty-five patients (10%) died before response assessment was performed (Table 3). CR/CRi rates were higher in patients with white blood cell (WBC) count ≤5 × 10^9^/L (46% vs 25%; *p* = 0.002) and platelet count >30 × 10^9^/L (46% vs 30%; *p* = 0.01), while all other characteristics did not associate with response (Supplementary Table S1).

**Table 3:** Treatment response of patients with relapsed/refractory AML treated with HMA/VEN (full cohort *N* = 240 and molecular cohort *n* = 174).

Parameter	HMA/VEN R/R (*N* = 240)	Molecular cohort (*n* = 174)
Best response, *n* (%)		
CR	60 (25)	42 (24)
CRi	33 (14)	26 (15)
MLFS	13 (5)	9 (5)
PR	22 (9)	–
SD/RD	87 (36)	77 (44)
Died before first assessment	25 (10)	20 (11)
Composite CR (CR, CRi)	93 (39)	68 (39)
ORR (CR, CR, MLFS)	106 (44)	77 (44)

*CR* complete remission, *CRi* complete remission with incomplete blood count recovery, *CRc* composite complete remission (CR+CRi), *MLFS* morphologic leukemia-free state, *ORR* overall response rate, *RD* refractory disease, *SD* stable disease.

**Fig. 1:**
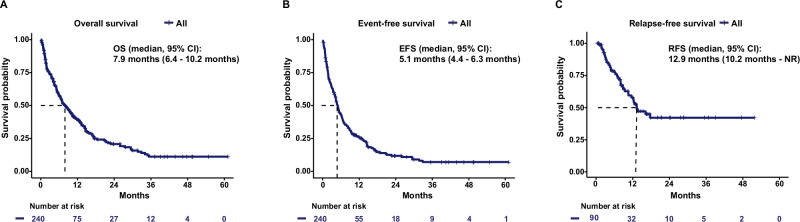
Kaplan–Meier estimates for survival for the entire cohort (*N* = 240). **A** Kaplan–Meier estimates for overall survival. **B** Kaplan–Meier estimates for event-free survival. **C** Kaplan–Meier estimates for relapse-free survival in CR/CRi patients.

After a median follow-up of 31.5 months, median OS was 7.9 months (95% CI, 6.4–10.2 months) and median EFS was 5.1 months (95% CI, 4.4–6.3 months) in the entire cohort. In CR/CRi patients median RFS was 12.9 months (95% CI, 10.2 months to not reached) (Fig. 1A–C).

### Clinical and molecular signatures of response

One-hundred-seventy-four patients with molecular data were evaluated for clinical and molecular predictors of response. In univariate analysis, CR/CRi was more likely to occur in patients with WBC count ≤5 × 10^9^/L (46% vs 20%; *p* = 0.02) and platelet count >30 × 10^9^/L (41% vs 29%; *p* = 0.05) (Supplementary Table S2).

Molecular predictors of inferior response included mutated *PTPN11* (mutated vs wildtype (wt) CR/CRi 0% vs 38%; *p* = 0.06). Mutated *NF1* and *TP53* were numerically associated with inferior response (*NF1* mutated vs wt CR/CRi 14% vs 37%; *p* = 0.2; *TP53* mutated vs wt CR/CRi 29% vs 38%; *p* = 0.36), whereas mutated *IDH2* (mutated vs wt CR/CRi 46% vs 34%; *p* = 0.2) or *STAG2* (mutated vs wt CR/CRi 53% vs 34%; *p* = 0.13) status were numerically associated with a higher rate of CR/CRi (Supplementary Table S3).

### Predictors of overall and event-free survival

We next evaluated clinical and molecular markers for associations with OS and EFS. In univariate analysis of pretreatment variables, predictors of inferior OS included age >70 years (HR 1.38 95% CI 1.0-1.98; *p* = 0.064), the presence of extramedullary disease (HR 2.06, 95% CI 1.33–3.2; *p* = 0.001), HMA pretreatment (HR 1.63, 95% CI 1.15–2.3; *p* = 0.006), higher WBC count (HR 1.38 95% CI 0.92–1.94; *p* = 0.12) and complex karyotype (HR 1.33, 95% CI 0.91-1.94; *p* = 0.14) (Supplementary Fig. S3, Supplementary Table S4). Predictors for inferior EFS further included the type of combination partner (decitabine vs. azacitidine: HR 1.48, 95% CI 1.02–2.13; *p* = 0.038). Molecular predictors of inferior OS included mutations in *NF1* (HR 2.53, 95% CI 1.2–5.4; *p* = 0.015), *PTPN11* (HR 2.26, 95% CI 1.5–3.4; *p* < 0.001), *TP53* (HR 1.5, 95% CI 0.96–2.3; *p* = 0.073), and *FLT3* (any *FLT3* mutation, HR 1.64, 95% CI 1.00–2.7; *p* = 0.05) (Supplementary Fig. S4, Supplementary Table S5). The number of prior treatment lines was not a significant parameter in univariate analysis for OS and EFS (Supplementary Table S5; Supplementary Fig. S5) and was not further considered. In multivariate analysis extramedullary disease (HR 2.46, 95% CI 1.5–4.0; *p* = <0.001), HMA pretreatment (HR 1.9, 95% CI 1.3-2.8; *p* < 0.001), presence of *PTPN11* (HR 2.2, 95% CI 1.3–4.0; *p* = 0.006), and *NF1* (HR 2.26, 95% CI 1.5–3.4; *p* < 0.001) mutations were identified as independent risk factors for inferior OS, whereas presence of *SF3B1* mutations (HR 0.38, 95% CI 0.2–0.8; *p* = 0.01) remained as independent risk factor for superior OS (Table 4). Independent risk factors for inferior EFS were similar to those for OS and included extramedullary disease (HR 1.76, 95% CI 1.1–2.8; *p* = 0.02), HMA pretreatment (HR 1.57, 95% CI 1.1–2.2; *p* = 0.014), and presence of mutations in *PTPN11* (HR 2.1, 95% CI 1.1–4.03; *p* = 0.03), and *FLT3* (HR 1.82, 95% CI 1.1–3.04; *p* = 0.02). Presence of a *SF3B1* mutation was associated with superior EFS (HR 0.42, 95% CI 0.2–0.8; *p* = 0.01) (Table 4).

**Table 4:** Uni- and multivariate analyses for overall survival (OS) and event-free survival (EFS) of molecular markers for OS and EFS in 174 acute myeloid leukemia (AML) patients.

Endpoint	Variables in the model	Univariate analysis			Multivariate analysis			
		HR^a^	95%CI	*p*	HR^a^	95%CI	*p*	Coefficient^b^
OS	Extramedullary AML	2.06	1.33;3.18	0.001	2.46	1.5;4.0	<0.001	0.90
	HMA pretreatment	1.63	1.15;2.31	0.006	1.9	1.3;2.8	<0.001	0.64
	*NF1* mut vs wt	2.53	1.19;5.35	0.015	2.6	1.2;5.4	0.01	0.94
	*PTPN11* mut vs wt	2.26	1.50;3.41	<0.001	2.2	1.3;4.0	0.006	0.80
	*FLT3*^c^ mut vs wt	1.64	1.00;2.68	0.05	1.6	1.0;2.8	0.08	0.49
	*TP53* mut vs wt	1.49	0.96;2.29	0.073	1.5	0.94;2.3	0.09	0.39
	*SF3B1* mut vs wt	0.56	0.31;1.02	0.058	0.38	0.2;0.8	0.01	-0.98
EFS	Extramedullary AML	1.62	1.04;2.53	0.032	1.76	1.1;2.8	0.02	0.57
	HMA pretreatment	1.46	1.04;2.05	0.028	1.57	1.1;2.2	0.014	0.45
	*NF1* mut vs wt	2.74	1.43;5.22	0.002	2.25	0.95;5.3	0.06	0.81
	*PTPN11* mut vs wt	1.95	1.14;3.36	0.016	2.1	1.1;4.03	0.03	0.73
	*FLT3*^c^ mut vs wt	1.97	1.25;3.11	0.003	1.82	1.1;3.04	0.02	0.6
	*SF3B1* mut vs wt	0.59	0.36;0.97	0.036	0.42	0.2;0.8	0.01	-0.87

For OS and EFS a model score value (S_OS/EFS_) < 0.25 corresponds to a favorable risk, 0.25 ≤ S_OS/EFS_ ≤ 0.75 to an intermediate risk and S_OS/EFS_ > 0.75 to an adverse risk.

*CI* confidence interval, *EFS* event-free survival, *HMA* hypomethylating agents (i.e. azacitidine, decitabine), *HR* hazard ratio, *p* *P*-value, *OS* overall survival.

^a^Hazard ratios greater than or less than 1 indicate an increased or decreased risk, respectively, of an event for the categories listed.

^b^The VEN-PRS is calculated by adding coefficients of all variables that apply for each individual patient.

^c^Any *FLT3* mutation. *FLT3* mutations n (%): *FLT3-*ITD: 18 (62); *FLT3-*TKD: 5 (17); atypical *FLT3*: 6 (21).

**Fig. 2:**
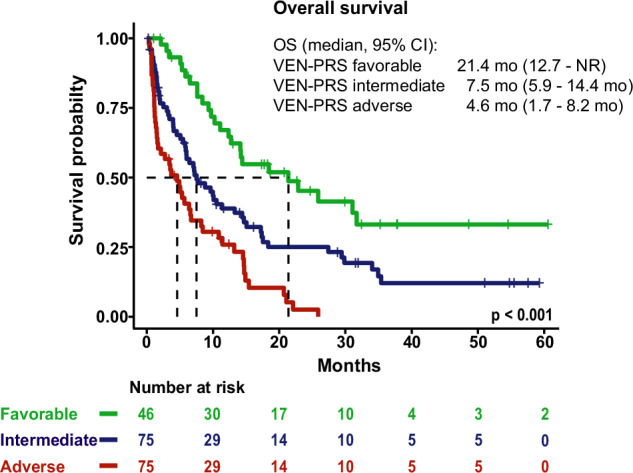
Kaplan–Meier estimates for overall survival of 174 patients with molecular information according to VEN-PRS classification. Favorable risk: S_OS_ < 0.25; intermediate risk: 0.25 ≤ S_OS_ ≤ 0.75; adverse risk: S_OS_ > 0.75.

Subsequently, a three-tiered risk stratification model was developed for OS and EFS based on HR-weighted scoring. For OS the following variables were included in the model: extramedullary AML, HMA pretreatment, mutated *PTPN11*, *FLT3*, *TP53*, *NF1*, and *SF3B1*. Each variable was weighted by calculating the hazard coefficient, which is the natural logarithm of the hazard ratio of the variable (Table 4). The individual score for each patient was calculated by adding up the coefficients of variables that apply for each individual patient. Based on the individual score, patients were stratified into three risk groups: patients with a VEN-PRS of <0.25 were assigned to the favorable risk group, patients with a VEN-PRS between 0.25 and 0.75 to the intermediate risk group, and patients with VEN-PRS > 0.75 to the adverse risk group (Supplementary Fig. S6A). When applying the newly developed VEN-PRS, it allocated 46 (26%), 75 (43%), and 53 patients (31%) into the favorable-, intermediate-, and adverse risk groups, respectively with significant differences in OS (median OS, 95% CI in favorable vs intermediate vs adverse risk groups: 14.2 months (10.2–31.1) vs 7.0 months (5.0–13.2) vs 3.1 months (1.5–6.7); *p* < 0.001) (Fig. 2).

**Fig. 3:**
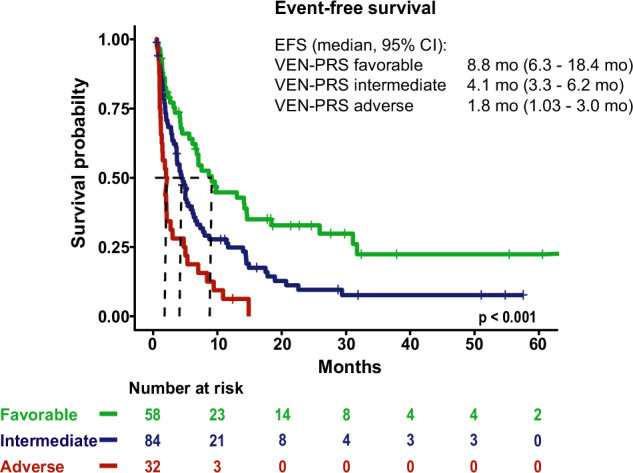
Kaplan–Meier estimates for event-free survival of 174 patients with molecular information according to VEN-PRS classification. Favorable risk: S_EFS_ < 0.25; intermediate risk: 0.25 ≤ S_EFS_ ≤ 0.75; adverse risk: S_EFS_ > 0.75.

For EFS the following variables were included to the model: extramedullary AML, HMA pretreatment, mutated *PTPN11*, *FLT3*, *NF1*, and *SF3B1*. Based on the individual scores patients were stratified into three risk groups for EFS analogous to OS (Supplementary Fig. S6B). The VEN-PRS model allowed to allocate patients into three risk groups with significantly different EFS (median EFS, 95% CI in favorable vs intermediate vs adverse risk group: 8.8 months (6.3–18.43) vs 4.1 months (3.33–6.2) vs 1.8 months (1.03–3.03); *p* < 0.001) (Fig. 3). One-, two-, and three-year OS and EFS are provided in Supplementary Table S6. Supplementary Table S7 provides the VEN-PRS calculator for estimating patient’s individual risk.

The VEN-PRS was evaluated separately in the relapsed and refractory cohorts and was similarly prognostic in these subgroups (Supplementary Figs. S7 and S8). The VEN-PRS was also prognostic in patients treated with HMA/VEN after one prior line of treatment for OS, but not EFS (Supplementary Fig. S9), and it was prognostic in patients treated with HMA/VEN after more than one prior line of treatment for both OS and EFS (Supplementary Fig. S10).

Calibration analysis showed that the predicted 10-month survival correlated well with the observed survival for the three risk groups, where EFS in the adverse risk group is somewhat underestimated, confirming that the model is well calibrated (Supplementary Fig. S11). In discrimination analysis, the AUC was 0.67 for OS and 0.68 for EFS, corresponding to a moderate discrimination (Supplementary Fig. S12).

As we did not have access to a second R/R AML cohort treated with venetoclax for external validation, we evaluated our score in patients treated with HMA/VEN at first diagnosis (*n* = 189). There were less patients in the adverse risk group since a significantly lower number of patients in the first-line cohort had prior HMA treatment (first-line *n* = 22; R/R AML *n* = 99) and extramedullary manifestation (first-line *n* = 4; R/R AML *n* = 17) (Supplementary Table S8), but the model showed significantly different risks for the favorable, intermediate and adverse risk groups for OS and for the favorable and intermediate risk groups for EFS, thus independently validating the VEN-PRS (Supplementary Figs. S13A and S13B). AIC differences are negative with -6.14 for OS and -6.94 for EFS, showing that our variable selection is suitable for modeling an independent cohort.

Taken together, our analyses suggest HMA pretreatment, extramedullary disease and the mutation status of *NF1, PTPN11*, *FLT3*, *TP53* and *SF3B1* as clinically relevant predictors of survival in patients treated with HMA/VEN for R/R AML.

### Comparison of VEN-PRS to other AML risk models

We first compared our prognostic score model with the prognostic index proposed by Breems and colleagues [12], which was developed to estimate the outcome of AML patients in first relapse. The prognostic index showed no significant fit to our data. The AIC difference between the prognostic index by Breems et al. and the null model was 3.72 and 3.97 for OS and EFS, respectively, whereas the AIC difference between our proposed model and the null model was −26.67 and −20.11 for OS and EFS, respectively. As a more negative AIC difference indicates less prognostic information loss, our model clearly outperforms the prognostic index by Breems (Supplementary Fig. S14).

We next evaluated the ELN2022 risk classification, which was developed for newly diagnosed AML patients undergoing intensive chemotherapy and thus is not expected to be prognostic in R/R patients [3]. CR/CRi rates were 42%, 44%, and 33% in favorable, intermediate, and adverse ELN2022 risk patients, respectively (*p* = 0.44). Overall survival in the context of ELN2022 in the favorable, intermediate, and adverse risk groups was 11.1 months, 8.4 months, and 9.3 months, respectively (*p* = 0.9). The AIC difference between the ELN2022 classification and the null model was 3.8 and 3.91 for OS and EFS, respectively.

We then evaluated the VIALE A prognostic score proposed by Döhner and colleagues [25], which stratified newly diagnosed HMA/VEN treated patients into three risk groups based on the mutational status of four genes (favorable: *TP53* wt, no *FLT3-*ITD*, K/NRAS* wt; intermediate *FLT3*-ITD or *K/NRAS* mutated; adverse *TP53* mutated) [24]. Median OS rates in favorable, intermediate, and adverse risk groups were 7.6 months, 12 months, and 8.2 months (*p* = 0.18), respectively, when applying the VIALE A prognostic score on our cohort of R/R AML patients (Supplementary Fig. S15). The AIC difference between the proposed VIALE A prognostic score and the null model was 0.90 and 0.32 for OS and EFS, respectively.

Lastly, we further compared our prognostic model with the recently published score by Gangat and colleagues, which stratified newly diagnosed patients undergoing HMA/VEN treatment into three risk groups based on HR-weighted scoring of the following parameters: failure to achieve CR/CRi, adverse karyotype, *TP53* mutation, and absence of *IDH2* mutation. Reported mOS were 28.9, 9.6, and 3.1 months for the low, intermediate and high-risk groups, respectively for AML patients treated with frontline HMA/VEN [26]. Applying the score on our R/R AML cohort, survival estimates in favorable, intermediate, and adverse risk groups were 24.1 months, 6.7 months, and 4.6 months (*p* < 0.001), respectively (Supplementary Fig. S16). While this model provided good separation in the R/R setting, the AIC difference between the proposed score by Gangat et al. classification and the null model was −16.03 for OS and thus performed less well compared to the proposed VEN-PRS model.

In summary, previously published risk scores and classifications showed significantly lower accuracy in predicting outcomes of R/R AML patients treated with HMA/VEN compared to our proposed risk model, suggesting that other risk factors than the previously established pre-treatment characteristics determine efficacy of HMA/VEN in R/R AML patients.

## Discussion

With HMA/VEN being increasingly used in relapsed/refractory AML patients, the evaluation of genetic and clinical signatures of response and survival is essential to support treatment selection for R/R AML patients. In this study, we analyzed a large cohort of relapsed or refractory AML patients treated with HMA/VEN and propose a novel prognostic risk score (VEN-PRS) derived from clinical and molecular parameters. When applying the VEN-PRS to R/R AML patients treated with HMA/VEN, patients were stratified into favorable (26%), intermediate (43%), and adverse risk (31%) groups. Outcome analysis demonstrated that the proposed VEN-PRS performed well, stratifying three different cohorts of patients with significant differences in overall and event-free survival, both in relapsed and refractory patients and patients with one or more than one prior lines of treatment.

This risk score identified extramedullary AML and HMA pretreatment as significant clinical variables and mutations in *NF1*, *PTPN11*, *FLT3*, *TP53*, and *SF3B1* as significant molecular markers for OS. Significant prognostic parameters for EFS included the aforementioned clinical and molecular variables except for *TP53*, which was not prognostic for EFS.

The approval of HMA/VEN marked a substantial improvement in the treatment landscape of elderly/unfit patients with newly diagnosed AML providing a new standard of care. However, long-term outcomes of this combination regimen remain to be determined, and with a third of patients being primary refractory and the majority of patients eventually relapsing, treatment is primarily of palliative intention [27, 28].

In our analysis a lower WBC count and a higher platelet count were associated with a higher CR/CRi rate, while mutations in *PTPN11, NF1, and TP53* were associated with a lower rate and mutations in *IDH2* and *STAG2* with a higher rate of CR/CRi. Molecular predictors of response to frontline HMA/VEN therapy include *NPM1, IDH1/2*, and *DNMT3A* mutations, whereas mutations in signaling genes (i.e. *FLT3-*ITD*, KRAS, NRAS, PTPN11, CBL, KIT)* and *TP53* were associated with secondary resistance and shorter survival [29]. Other molecular markers that are associated with response outcomes include mutations in *CEBPA*, *SRSF2* and *ASXL1* [19, 30]. Shimony et al. recently reported on the clinical impact of molecular ontogeny in a large cohort of newly diagnosed AML patients who were treated with either HMA and venetoclax or HMA monotherapy. In patients with *TP53* mutated AML, mOS and response rates were similar in patients treated with HMA/VEN vs HMA monotherapy. Patients with secondary ontogeny, defined by the presence of mutations in *ASXL1*, *BCOR*, *EZH2*, *SF3B1*, *SRSF2*, *STAG2*, *U2AF1*, or *ZRSR2*, showed prolonged OS and better response to HMA/VEN as compared to HMA monotherapy. However, in our study, OS for patients with secondary ontogeny was similar to what was observed for patients with de novo disease [31]. In line with our results, another study demonstrated that the addition of VEN to low intensity chemotherapy abrogated the adverse risk of splicing mutations [32]. Stahl et al. reported higher response rates in *NPM1* mutated R/R AML patients and adverse cytogenetics. Mutations in *TP53, KRAS/NRAS*, and *SF3B1* were associated with worse OS [16]. However, in other reports by Morsia et al. and Piccini et al., abnormal cytogenetics did not predict response or survival in HMA/VEN treated R/R AML patients [17, 18]. In another retrospective analysis by Aldoss et al. mutations in *TET2* and *ASXL1* were associated with improved response while responses were similar in *TP53* and *FLT3* wt and mutated patients [19]. Real-world data from the French Auraml Group demonstrated response rates of up to 63% in IDH mutated R/R AML patients [20].

Two recently published communications from the Mayo Clinic and MD Anderson Cancer Center proposed genetic based risk scores to predict response and survival outcomes in newly-diagnosed patients treated with upfront HMA/VEN therapy [26, 33]. The 4-gene molecular prognostic risk signature from MD Anderson Cancer Center uses *N/KRAS, FLT3*-ITD and *TP53* mutations to stratify patients into three risk groups [33], whereas the score proposed by Gangat et al. integrates ELN2022 adverse karyotype and failure to achieve CR/CRi in addition to molecular markers in their score [26]. Our findings of the differential benefit of HMA/VEN in R/R AML by molecular subgroups correspond with the aforementioned studies, which identified *TP53, PTPN11* and *FLT3* as adverse prognostic risk markers. Further, *SF3B1* was identified as a favorable risk parameter, whereas other secondary ontogeny markers were not considered as adverse risk. While *TP53* was found to be prognostically relevant for OS in our analysis, it was not prognostic for EFS. Our observation is supported by subgroup analyses from the VIALE A trial and other studies of upfront HMA/VEN treatment, which show that *TP53* mutated patients respond quite well to HMA/VEN but do not derive long-term benefit from addition of VEN [27, 34, 35]. However, the *TP53* mutation is found to be one of the main molecular denominators of secondary resistance and relapse resulting in a general dismal prognosis in *TP53* mutated AML irrespective of treatment modality [36, 37]. Importantly, the VEN-PRS is applicable at the time of relapse and refractoriness and does not rely on response to salvage treatment.

Widely applied risk scores including the ELN2022 classification are effective in stratifying intensively treated patients according to molecular and cytogenetic risk at diagnosis. As the ELN2022 classification is derived from data of patients who were treated with intensive, mostly anthracycline based therapies, it is not expected to predict prognosis of patients treated with HMA/VEN and other lower-intensity regimens.

The VEN-PRS requires a medical history, physical examination and possibly imaging to detect extramedullary disease, and molecular profiling at time of relapse or refractoriness. We provide an easily applicable calculator for estimating patients’ individual risk based on globally accessible parameters that can support therapeutic decision-making regarding HMA/VEN versus alternative treatment options (VEN-PRS calculator). More and more frequently patients with R/R AML have several treatment options such as inhibitors of *FLT3, IDH1/2*, or *menin*, in addition to HMA/VEN, especially for relapse after alloHCT. Choosing among treatment options is difficult in this setting as no comparative studies are available. Prognostic risk scores like the one proposed here will therefore help to estimate prognosis under HMA/VEN treatment, and will facilitate the discussion with patients to meet an informed treatment decision.

Limitations of the current study include its design as a registry, which does not control for a selection bias, and a limited number of patients, which limit the evaluation of genetic markers to the more frequently mutated subgroups.

In conclusion, the current study identifies predictors of survival to HMA/VEN in R/R AML and proposes a clinical-molecular survival model, which is practically relevant and allows a clear stratification of patients into three risk groups with significantly different OS and EFS outcomes. Our proposed VEN-PRS is an efficiently designed prognostic tool to support informed therapeutic decision in this difficult-to-treat population.

## Supplementary information

VEN-PRS_Supplemental Table S7

VEN-PRS_Supplementary Material

## Data Availability

Individual patient data will not be made available in order to maintain health information privacy. De-identified mutation information will be shared upon reasonable request to the corresponding author.
