# Supplementary material for: A novel prognostic risk model for patients with refractory/relapsed acute myeloid leukemia receiving venetoclax plus hypomethylating agents
Source: Leukemia. 2025 Jan 8;39(3):614–22. doi: 10.1038/s41375-024-02501-6 (PMC11879869; doi:10.1038/s41375-024-02501-6)
Supplement: Supplementary file 2 — VEN-PRS_Supplementary Material [file 41375_2024_2501_MOESM2_ESM.docx]

# **A Novel Prognostic Risk Model for Patients with Refractory/Relapsed Acute Myeloid Leukemia Receiving Venetoclax plus Hypomethylating Agents**

Rabia Shahswar^1^, Razif Gabdoulline^1^, Katja Krueger^1^, Martin Wichmann^1^, Katharina S. Götze^2,3^, Krischan Braitsch^2^, Manja Meggendorfer^4^, Laura Schmalbrock^5^, Lars Bullinger^5^, Franziska Modemann^,6^, Walter Fiedler^6^, Juergen Krauter^7^, Stephan Kaun^8^, Susanne Rotermund^8^, Andreas Voß^9^, Yvonne Lisa Behrens^10^, Anke Katharina Bergmann^10^, Elisabeth Koller^11^, Gernot Beutel^1^, Felicitas Thol^1^, Florian Heidel^1^, Michael Heuser^1,12^

# **Supplemental Materials**

## **Supplemental Methods**

### Cytogenetic and Molecular Analysis

Molecular and cytogenetic analysis was performed centrally by next-generation sequencing (NGS) and G- and R-banding analysis using peripheral blood or bone marrow as reported previously. (1) Mutations associated with myeloid leukemias were detected using a custom TruSight or Nextera myeloid sequencing panel, which included 46 and 48 genes, respectively. (Illumina, San Diego, CA). Samples were sequenced on a MiSeq sequencer and sequencing data was analyzed as described previously using a variant allele frequency cutoff of 5%.(1) Molecular analysis was performed before start of VEN-based therapy. One-hundred-and-forty-three patients had available karyotype data at the time of R/R AML before start of HMA/VEN, while 62 patients had available data from the time of first diagnosis, and 37 patients had no cytogenetic data available.

### Statistical Analysis

Treatment response and outcome measures were assessed according to 2022 ELN criteria. (2) Besides complete remission (CR) and complete remission with incomplete blood count recovery (CRi) we also report the overall response rate (ORR), which includes CR/CRi and morphologic leukemic free state (MLFS; defined as less than 5% blasts in an aspirate sample without hematological recovery).(2) Patients with delayed recovery of neutrophils and platelets were considered in CR, if neutrophils and platelets recovered before the next course of treatment and blasts were below 5% in bone marrow and absent in peripheral blood. Blasts in bone marrow ≥5%, persisting blasts in peripheral blood, or extramedullary disease at time of response assessment was defined as refractory disease (RD). Timing of response evaluation was at the discretion of the treating physician; bone marrow biopsy was obtained after the completion of either one or two cycles in the majority of cases. Outcome measures included overall survival (OS), event free survival (EFS), and relapse-free survival (RFS), and were analyzed as previously reported.(2, 3)

Demographic characteristics were analyzed as previously reported.(4) Univariate and multivariate Cox proportional hazards models were used to evaluate the impact of relevant clinical and genetic variables on OS and EFS. A multivariate model was built including variables with *p*<0.15 in univariate analysis and at least 6 patients with the aberrant variable (see Supplemental Tables S5 and S6). The backward elimination procedure was performed with the worst significance of fit until the maximum value of the Akaike information criterion (AIC) difference from the null model was achieved. The AIC estimates the relative amount of information lost by a given model. The best model from the set of plausible models being considered is therefore the one with the lowest AIC value (the least information loss relative to the true model).(5)

The VEN-PRS was determined for all patients, and the distribution of scores in the cohort was evaluated. Given the observed clustering of the VEN-PRS (**Supplemental Figure S6**), some clusters were combined to form three similarly sized cohorts. A formal approach was employed to identify the optimal clustering of data using the Gaussian mixture model.(6) The best model according to the Bayesian Information Criterion (BIC) identified 7 clusters for OS (**Supplemental Figure S6A**) and 4 clusters for EFS (**Supplemental Figure S6B**). These clusters were similarly merged to form three groups with the following cut-offs for OS and EFS: S_OS/EFS_ < 0.25 for the low-risk cohort, 0.25 < S_OS/EFS_ ≤ 0.75 for the intermediate-risk cohort, and S_OS/EFS_ > 0.75 for the adverse-risk cohort.

### **Supplemental Tables**

### **Supplemental Table S1:** Univariate analysis of clinical characteristics for CR/CRi in 240 AML patients (entire cohort).

| **Clinical characteristics** | **CR/CRi, n** | **CR/CRi rates** | **OR (95% CI)** | ***p*** |
| --- | --- | --- | --- | --- |
| Age > 70 years vs ≤70 years | 43/101 vs. 46/126 | 43% vs. 37% | 1.1 (0.7-2.0) | 0.35 |
| Male vs female sex | 57/138 vs. 36/102 | 41% vs. 35% | 1.29 (0.76-2.2) | 0.35 |
| sAML/tAML vs de novo AML | 38/107 vs. 55/133 | 36% vs. 41% | 0.78 (0.46-1.32) | 0.36 |
| ICC 2022 diagnostic qualifiers, n (%)  with vs without diagnostic qualifiers* | 40/115 vs. 53/125 | 35% vs. 42% | 0.72 (0.43-1.2) | 0.23 |
| Time to relapse  7-18 mo vs ≤ 6 months  > 18 mo vs ≤ 6 months | 9/23 vs. 8/21  8/15 vs. 8/21 | 39% vs. 38%  53% vs. 38% | 1.04 (0.3-3.5)  1.86 (0.48-7.1) | 0.94  0.36 |
| Complex karyotype yes vs no | 21/70 vs. 58/135 | 30% vs. 43% | 0.57 (0.3-1.05) | 0.2 |
| ELN2022  Adverse vs favorable  Intermediate vs favorable | 48/136 vs. 5/12  23/47 vs. 5/12 | 35% vs. 42%  49% vs. 42% | 0.76 (0.23-2.55)  1.34 (0.37-4.83) | 0.66  0.56 |
| Extramedullary AML yes vs no | 7/19 vs. 85/218 | 37% vs. 39% | 0.92 (0.35-2.4) | 0.9 |
| Prior alloHCT yes vs no | 33/83 vs. 55/148 | 40% vs. 37% | 1.1 (0.64-1.94) | 0.5 |
| Prior intensive chemotherapy yes vs no | 62/162 vs. 31/78 | 38% vs. 40% | 0.94 (0.54-1.63) | 0.83 |
| Prior HMA treatment yes vs no | 44/127 vs. 49/112 | 35% vs. 44% | 0.68 (0.4-1.15) | 0.26 |
| WBC before VEN >5x10^9^/L vs ≤5x10^9^/L | 17/69 vs. 73/158 | 25% vs. 46% | 0.6 (0.35-1.0) | **0.002** |
| Hemoglobin before VEN >9g/dL vs ≤9g/dL | 50/110 vs. 39/117 | 45% vs. 33% | 1.5 (0.86-2.5) | 06 |
| Platelet count before VEN >30x10^9^/L vs ≤30x10^9^/L | 57/123 vs. 31/103 | 46% vs. 30% | 1.9 (1.1-3.3) | **0.01** |
| Decitabine vs azacitidine | 14/48 vs. 79/192 | 29% vs. 41% | 0.6 (0.3-1.17) | 0.13 |
| Other VEN duration vs 14 days | 44/110 vs. 46/121 | 40% vs. 38% | 1.1 (0.64-1.85) | 0.76 |

Abbreviations: alloHCT, allogeneic hematopoietic cell transplantation; CR, complete remission; CRi, complete remission with incomplete blood count recovery; ELN, European LeukemiaNet; HMA, hypomethylating agents (i.e. azacitidine, decitabine), mo, months; *p*, P-value; sAML, secondary AML; t-AML, therapy-related AML; VEN, venetoclax; WBC, white blood count.

*ICC^1^ diagnostic qualifiers: therapy-related; progressed from MDS; progressed from MDS/MPN; germline predisposition.

### **Supplemental Table S2:** Univariate analysis of clinical characteristics for CR/CRi in 174 AML patients (molecular cohort).

| **Clinical characteristics** | **CR/CRi, n** | **CR/CRi rates** | **OR (95% CI)** | ***p*** |
| --- | --- | --- | --- | --- |
| Age > 70 years vs ≤70 years | 28/82 vs 35/92 | 34% vs 38% | 0.79 (0.42;1.47) | 0.53 |
| Male vs female sex | 37/99 vs 26/75 | 37% vs 35% | 1.12 (0.60;2.11) | 0.75 |
| ICC 2022 diagnostic qualifiers, n (%)  with vs without diagnostic qualifiers* | 28/85 vs 35/89 | 33% vs 39% | 0.74 (0.40;1.38) | 0.43 |
| Relapsed vs refractory AML | 31/78 vs 32/96 | 40% vs 33% | 1.06 (0.55;2.04) | 0.45 |
| Time to relapse (Breems et al.)  7-18 mo vs ≤ 6 months  > 18 mo vs ≤ 6 months | 8/21 vs 8/19  6/11 vs 8/19 | 38% vs 42%  55% vs 42% | 0.46 (0.06;2.63)  1.38 (0.41;4.73) | 0.56** |
| Complex karyotype yes vs. no | 13/50 vs 41/107 | 26% vs 38% | 0.57 (0.26;1.17) | 0.15 |
| ELN2022  Adverse vs favorable  Intermediate vs favorable | 40/120 vs 5/12  18/41 vs 5/12 | 33% vs 42%  44% vs 42% | 0.7 (0.21;2.49)  1.1 (0.3;4.25) | 0.43** |
| Extramedullary AML yes vs no | 6/17 vs 57/157 | 35% vs 36% | 0.96 (0.32;2.66) | 1.00 |
| AlloHCT before VEN yes vs no | 25/58 vs 37/115 | 43% vs 32% | 1.6 (0.83;3.06) | 0.18 |
| Intensive pretreatment yes vs no | 41/109 vs 22/65 | 38% vs 34% | 1.18 (0.62;2.26) | 0.63 |
| HMA pretreatment yes vs no | 32/99 vs 31/75 | 32% vs 41% | 0.68 (0.36-1.26) | 0.27 |
| WBC before VEN ≤5x10^9^/L vs >5x10^9^/L | 52/113 vs 10/51 | 46% vs 20% | 2.38 (1.2;4.76) | **0.02** |
| Hemoglobin before VEN >9g/dL vs ≤9g/dL | 30/71 vs 17/64 | 42% vs 27% | 1.37 (0.73;2.61) | 0.34 |
| Platelet count before VEN >30x10^9^/L vs ≤30x10^9^/L | 41/99 vs 22/75 | 41% vs 29% | 1.92 (1.01;3.72) | **0.05** |
| Decitabine vs azacitidine | 11/34 vs 51/137 | 32% vs 37% | 0.56 (0.25;1.19) | 0.19 |
| Other VEN duration vs 14 days | 27/84 vs 36/90 | 32% vs 40% | 0.69 (0.36;1.32) | 0.33 |

Abbreviations: alloHCT, allogeneic hematopoietic cell transplantation; CR, complete remission; CRi, complete remission with incomplete blood count recovery; ELN, European LeukemiaNet; HMA, hypomethylating agents (i.e. azacitidine, decitabine), mo, months; *p*, P-value; sAML, secondary AML; t-AML, therapy-related AML; VEN, venetoclax; WBC, white blood count.

*ICC^1^ diagnostic qualifiers: therapy-related; progressed from MDS; progressed from MDS/MPN; germline predisposition.

**Grouped P-value.

### **Supplemental Table S3:** Univariate analysis of molecular markers for CR/CRi in 174 AML patients.

| **Molecular markers*** | **CR/CRi, n** | **CR/CRi rates** | **OR (95% CI)** | ***p*** |
| --- | --- | --- | --- | --- |
| *ASXL1* mut vs wt | 10/32 vs 53/142 | 31% vs 37% | 0.76 (0.32;1.70) | 0.52 |
| *BCOR* mut vs wt | 11/21 vs 52/153 | 52% vs 34% | 2.14 (0.85;5.45) | 0.1 |
| *BCORL1* mut vs wt | 5/9 vs 58/165 | 56% vs 35% | 2.31 (0.59;9.63) | 0.2 |
| *CEBPA* mut vs wt | 4/13 vs 59/161 | 31% vs 37% | 0.77 (0.20;2.47) | 0.7 |
| *DDX41* mut vs wt | 1/6 vs 62/168 | 17% vs 37% | 0.34 (0.02;2.18) | 0.3 |
| *DNMT3A* mut vs wt | 11/29 vs 52/145 | 38% vs 36% | 1.09 (0.47;2.46) | 0.83 |
| *EZH2* mut vs wt | 2/6 vs 61/168 | 33% vs 36% | 0.88 (0.12;4.63) | 0.9 |
| *FLT3*** mut vs wt | 7/27 vs 56/147 | 26% vs 38% | 0.57 (0.21;1.38) | 0.23 |
| *FLT3*-ITD mut vs wt | 7/18 vs 56/156 | 39% vs 36% | 1.26 (0.44;3.47) | 0.65 |
| *GATA2* mut vs wt | 1/7 vs 62/167 | 14% vs 37% | 0.28 (0.01;1.70) | 0.2 |
| *IDH1* mut vs wt | 4/13 vs 59/161 | 31% vs 37% | 0.77 (0.20;2.47) | 0.67 |
| *IDH2* mut vs wt | 13/28 vs 50/146 | 46% vs 34% | 1.66 (0.73;3.78) | 0.2 |
| *JAK2* mut vs wt | 4/9 vs 59/165 | 44% vs 36% | 1.44 (0.34;5.63) | 0.6 |
| *NF1* mut vs wt | 1/7 vs 62/167 | 14% vs 37% | 0.28 (0.01;1.70) | 0.2 |
| *NPM1* mut vs wt | 8/18 vs 55/156 | 44% vs 35% | 1.47 (0.53;3.94) | 0.44 |
| *NRAS* mut vs wt | 4/17 vs 59/157 | 24% vs 38% | 0.51 (0.14;1.52) | 0.25 |
| *PHF6* mut vs wt | 2/7 vs 61/167 | 29% vs 37% | 0.70 (0.10;3.33) | 0.7 |
| *PTPN11* mut vs wt | 0/6 vs 63/168 | 0% vs 38% | 0 (NA) | 0.06 |
| *RAD21* mut vs wt | 4/8 vs 59/166 | 50% vs 36% | 1.81 (0.42;7.92) | 0.4 |
| *RUNX1* mut vs wt | 13/34 vs 50/140 | 38% vs 36% | 1.11 (0.50;2.39) | 0.78 |
| *SF3B1* mut vs wt | 4/7 vs 59/167 | 57% vs 35% | 2.44 (0.52;12.74) | 0.2 |
| *SRSF2* mut vs wt | 11/27 vs 52/147 | 41% vs 35% | 1.26 (0.53;2.89) | 0.59 |
| *STAG2* mut vs wt | 9/17 vs 54/157 | 53% vs 34% | 2.15 (0.78;6.02) | 0.13 |
| *TET2* mut vs wt | 15/36 vs 48/138 | 42% vs 35% | 1.34 (0.62;2.82) | 0.44 |
| *TP53* mut vs wt | 9/31 vs 54/143 | 29% vs 38% | 0.67 (0.28;1.53) | 0.36 |
| *WT1* mut vs wt | 3/12 vs 60/162 | 25% vs 37% | 0.57 (0.12;1.98) | 0.4 |

*Genes mutated in 6 or more patients.

**Any *FLT3* mutation: (n, %): *FLT3*-ITD: 18 (62); *FLT3*-TKD: 5 (17); atypical *FLT3*: 6 (21).

Abbreviations: CR, complete remission; CRi, complete remission with incomplete blood count recovery; mut, mutated; *p*, P-value; wt, wildtype.

### **Supplemental Table S4:** Univariate analysis of clinical characteristics for OS and EFS in the molecular cohort of 174 AML patients.

| **Variables in the model** | **OS** | | |  | **EFS** | | |
| --- | --- | --- | --- | --- | --- | --- | --- |
|  | **HR^$^** | **95%CI** | ***p*** |  | **HR^$^** | **95%CI** | ***p*** |
| **Clinical characteristics** |  |  |  |  |  |  |  |
| Age > 70 years vs ≤70 years | 1.38 | 1.0;1.98 | 0.064 |  | 1.09 | 0.79;1.52 | 0.6 |
| Male vs female sex | 1.04 | 0.73;1.46 | 0.84 |  | 0.89 | 0.64;1.24 | 0.48 |
| ICC 2022 diagnostic qualifiers, n (%)  with vs without diagnostic qualifiers* | 1.19 | 0.85;1.67 | 0.32 |  | 1.18 | 0.85;1.64 | 0.33 |
| Relapsed vs refractory AML | 1.06 | 0.73;1.52 | 0.77 |  | 1.08 | 0.76;1.53 | 0.66 |
| Time to relapse (Breems et al.)  7-18 mo vs ≤ 6 months  > 18 mo vs ≤ 6 months | 1.15  0.78 | 0.5;2.84  0.4;1.56 | 0.77  0.48 |  | 1.58  0.95 | 0.65;3.89  0.49;1.83 | 0.31  0.87 |
| Number of prior treatment lines  2 or more prior lines vs. 1 prior line | 1.08 | 0.72;1.63 | 0.76 |  | 1.1 | 0.73;1.64 | 0.64 |
| Complex karyotype yes vs no | 1.33 | 0.91;1.94 | **0.14^#^** |  | 1.43 | 1.00;2.04 | **0.05^#^** |
| ELN2022  Adverse vs favorable  Intermediate vs favorable | 0.9  0.85 | 0.44;1.81  0.39;1.82 | 0.8  0.7 |  | 1.10  1.12 | 0.55; 2.23  0.53; 2.38 | 0.79  0.77 |
| Extramedullary AML  Yes vs no | 2.06 | 1.33;3.2 | **0.001^#^** |  | 1.62 | 1.04;2.53 | **0.032^#^** |
| AlloHCT before VEN  Yes vs no | 0.84 | 0.58;1.22 | 0.37 |  | 0.98 | 0.69;1.39 | 0.89 |
| Intensive pretreatment  Yes vs no | 0.73 | 0.52;1.01 | **0.059^#^** |  | 0.84 | 0.62;1.16 | 0.3 |
| HMA pretreatment yes vs no | 1.63 | 1.15;2.3 | **0.006^#^** |  | 1.46 | 1.04;2.05 | **0.028^#^** |
| WBC before VEN >5x10^9^/L vs ≤5x10^9^/L | 1.34 | 0.92;1.94 | **0.12^#^** |  | 1.26 | 0.89;1.78 | 0.19 |
| Hemoglobin before VEN >9g/dL vs ≤9g/dL | 0.79 | 0.56;1.13 | 0.2 |  | 0.84 | 0.6;1.18 | 0.33 |
| Platelet count >30x10^9^/L vs ≤30x10^9^/L | 0.83 | 0.58;1.19 | 0.31 |  | 0.9 | 0.64;1.27 | 0.55 |
| Decitabine vs azacitidine | 1.27 | 0.87;1.85 | 0.21 |  | 1.48 | 1.02;2.13 | **0.038^#^** |
| Other VEN duration vs 14 days | 1.03 | 0.72;1.48 | 0.86 |  | 1.01 | 0.72;1.44 | 0.94 |

Abbreviations: alloHCT, allogeneic hematopoietic cell transplantation; CI, confidence interval, EFS, event-free survival; ELN, European LeukemiaNet ;HMA, hypomethylating agents (i.e. azacitidine, decitabine), HR, hazard ratio; *p*, P-value; OS, overall survival; sAML, secondary AML; t-AML, therapy-related AML; VEN, venetoclax; WBC, white blood count.

**^$^**Hazard ratios greater than or less than 1 indicate an increased or decreased risk, respectively, of an event for the first category listed.

*ICC^1^ diagnostic qualifiers: therapy-related; progressed from MDS; progressed from MDS/MPN; germline predisposition.

^#^ Parameters with *p*<0.15 in univariate analysis and at least 6 patients with the aberrant variable, that were included in the multivariate analysis.

### **Supplemental Table S5:** Univariate analysis of molecular markers for OS and EFS in the molecular cohort of 174 AML patients.

| **Variables in the model** | **OS** | | |  | **EFS** | | |
| --- | --- | --- | --- | --- | --- | --- | --- |
|  | **HR^$^** | **95%CI** | ***p*** |  | **HR^$^** | **95%CI** | ***p*** |
| **Molecular markers*** |  |  |  |  |  |  |  |
| *ASXL1* mut vs wt | 0.95 | 0.6;1.49 | 0.83 |  | 0.9 | 0.57;1.41 | 0.65 |
| *BCOR* mut vs wt | 0.84 | 0.53;1.31 | 0.44 |  | 0.74 | 0.49;1.11 | 0.15 |
| *BCORL1* mut vs wt | 0.96 | 0.46;2.01 | 0.92 |  | 0.78 | 0.36;1.68 | 0.52 |
| *CEBPA* mut vs wt | 0.97 | 0.42;2.21 | 0.93 |  | 1.01 | 0.49;2.07 | 0.97 |
| *DDX41* mut vs wt | 1.10 | 0.52;2.31 | 0.8 |  | 1.25 | 0.73;2.16 | 0.42 |
| *DNMT3A* mut vs wt | 0.82 | 0.51;1.34 | 0.44 |  | 0.80 | 0.49;1.30 | 0.36 |
| *EZH2* mut vs wt | 1.42 | 0.46;4.37 | 0.54 |  | 1.15 | 0.37;3.55 | 0.81 |
| *FLT3*** mut vs wt | 1.64 | 1.00;2.7 | **0.05^#^** |  | 1.97 | 1.25;3.11 | **0.003^#^** |
| *FLT3-*ITD mut. vs wt | 1.23 | 0.74;2.1 | 0.43 |  | 1.7 | 1.1;2.5 | **0.02^#^** |
| *GATA2* mut vs wt | 0.77 | 0.33;1.80 | 0.55 |  | 0.89 | 0.38;2.08 | 0.8 |
| *IDH1* mut vs wt | 0.79 | 0.41;1.52 | 0.49 |  | 0.87 | 0.45;1.65 | 0.66 |
| *IDH2* mut vs wt | 0.99 | 0.61;1.61 | 0.96 |  | 0.82 | 0.52;1.30 | 0.39 |
| *JAK2* mut vs wt | 1.49 | 0.73;3.05 | 0.27 |  | 1.10 | 0.56;2.20 | 0.78 |
| *KRAS* mut vs wt | 1.53 | 0.85;2.75 | 0.15 |  | 1.17 | 0.57;2.42 | 0.66 |
| *NF1* mut vs wt | 2.53 | 1.2;5.4 | **0.015^#^** |  | 2.74 | 1.43;5.22 | **0.002^#^** |
| *NPM1* mut vs wt | 1.33 | 0.72;2.47 | 0.36 |  | 1.05 | 0.57;1.93 | 0.89 |
| *NRAS* mut vs wt | 0.98 | 0.68;1.41 | 0.89 |  | 1.16 | 0.87;1.55 | 0.31 |
| *NRAS/KRAS* mut vs wt | 1.09 | 0.78;1.54 | 0.6 |  | 1.15 | 0.85;1.57 | 0.36 |
| *PHF6* mut vs wt | 1.37 | 0.83;2.25 | 0.22 |  | 1.48 | 0.79;2.78 | 0.22 |
| *PTPN11* mut vs wt | 2.26 | 1.5;3.4 | **<0.001^#^** |  | 1.95 | 1.14;3.36 | **0.016^#^** |
| *RAD21* mut vs wt | 1.60 | 0.86;2.98 | 0.14 |  | 1.18 | 0.68;2.05 | 0.55 |
| *RUNX1* mut vs wt | 0.91 | 0.60;1.41 | 0.68 |  | 0.97 | 0.63;1.49 | 0.9 |
| *SF3B1* mut vs wt | 0.56 | 0.31;1.02 | **0.058^#^** |  | 0.59 | 0.36;0.97 | **0.036^#^** |
| *SRSF2* mut vs wt | 0.87 | 0.53;1.41 | 0.56 |  | 0.86 | 0.52;1.40 | 0.54 |
| *STAG2* mut vs wt | 0.56 | 0.31;1.00 | **0.053^#^** |  | 0.63 | 0.37;1.09 | **0.09^#^** |
| *TET2* mut vs wt | 1.13 | 0.78;1.63 | 0.52 |  | 1.00 | 0.68;1.47 | 0.98 |
| *TP53* mut vs wt | 1.49 | 0.96;2.29 | **0.073^#^** |  | 1.44 | 0.94;2.21 | **0.098^#^** |

Abbreviations: CI, confidence interval, EFS, event-free survival; HR, hazard ratio; *p*, P-value; OS, overall survival.

**^$^**Hazard ratios greater than or less than 1 indicate an increased or decreased risk, respectively, of an event for the category listed.

*Genes mutated in 6 or more patients.

**Any *FLT3* mutation. *FLT3* mutations n (%): *FLT3-*ITD: 18 (62); *FLT3*-TKD: 5 (17); atypical FLT3: 6 (21).

**^#^**Parameters with *p*<0.15 in univariate analysis and at least 6 patients with the aberrant variable, that were included in the multivariate analysis.

### **Supplemental Table S6:** One-, two-, and three year-survival outcomes of 174 patients with molecular information according to VEN-PRS classification.

|  | **OS** | | |  | **EFS** | | |
| --- | --- | --- | --- | --- | --- | --- | --- |
|  | **1 year** | **2 year** | **3 year** |  | **1 year** | **2 year** | **3 year** |
| **VEN-PRS** |  |  |  |  |  |  |  |
| Favorable | 62% | 44% | 29% |  | 45% | 32% | 19% |
| Intermediate | 39% | 25% | 12% |  | 25% | 9% | 7% |
| Adverse | 26% | 3% | 0% |  | 6% | 0% | 0% |

### **Supplemental Table S7:** VEN-PRS calculator to estimate a patient’s individual risk. The calculator is provided as a separate excel file.

### **Supplemental Table S8:** Demographic and disease characteristics of patients with AML receiving HMA/VEN for first-line treatment (validation cohort).

| **Patient and disease characteristics** | **Entire cohort (N=189)** |
| --- | --- |
| Age  Median (years, range) | 76 (27-91) |
| Sex, n (%)  Male  Female | 126 (67)  63 (33) |
| ECOG, n (%)  0  1  ≥ 2  Missing | 11 (6)  68 (36)  53 (28)  57 (30) |
| ELN 2022 risk group, n (%)  Favorable/ Intermediate  Adverse  Missing | 65 (34)  123 (65)  1 (0,5) |
| Complex karyotype, n (%)  Yes  No  Missing | 38 (20)  126 (67)  25 (13) |
| Extramedullary disease, n (%)  Yes  No  Missing | 4 (2)  180 (95)  5 (3) |
| Duration of VEN (per cycle), n (%)  28d  Other  Missing | 91 (48)  85 (45)  13 (7) |
| Dose: ramp up, n (%)  Yes  No  Missing | 99 (52)  82 (43)  8 (4) |
| First combination partner, n (%)  Azacitidine  Decitabine  Missing | 163 (86)  23 (12)  3 (2) |
| Azoles  Yes  No  Missing | 90 (48)  79 (42)  20 (11) |
| Prior HMA treatment, n (%)  Yes  No  Missing | 22 (12)  164 (87)  3 (2) |
| Mutations, n (%)  Cohesin complex  Epigenetic modifiers  Nucleophosmin  Signal transduction  Spliceosome  Transcription factor  Tumor suppressor  No mutation | 22 (12)  124 (66)  42 (22)  93 (49)  43 (23)  52 (28)  35 (19)  11 (6) |
| Overall response, n (%)  Yes  No  Missing | 86 (46)  73 (39)  30 (16) |

## **Supplemental Figures**

All AML R/R patients in VENReg **(n=341)**

Excluded patients **(n=74)**

- Intensive treatment regimen (n=72)
- Monotherapy (n=2)

R/R patients receiving VEN-based non-intensive chemotherapy **(n=267)**

Excluded patients **(n=9)**

- LDAC (n=7)
- Other non-intensive regimens (n=2)

R/R patients receiving VEN+HMA **(n=258)**

Response

- No data on response **(n=18)**

**Entire cohort (n=240)**

Molecular data

- No data on molecular genetics **(n=66)**

**Molecular cohort (n=174)**

**Supplemental Figure S1:** CONSORT diagram depicting patient selection. Patients with available response and survival data represent the entire cohort (N=240), and patients with additional molecular data constitute the molecular cohort (n=174).


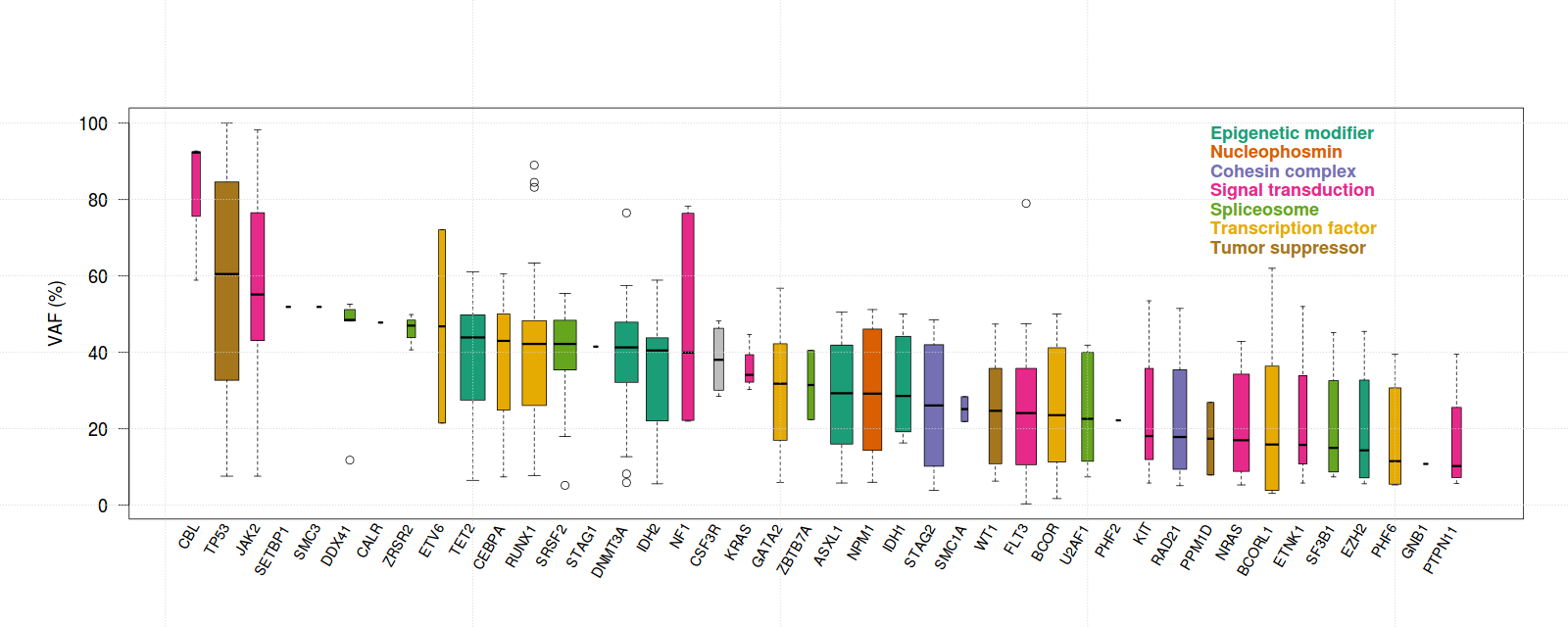
**Supplemental Figure S2:** Variant allele frequency (VAF) of mutations by gene. Median VAF is represented by a horizontal bar. Whiskers represent the interquartile range. The width of the bars represents the relative number of patients.


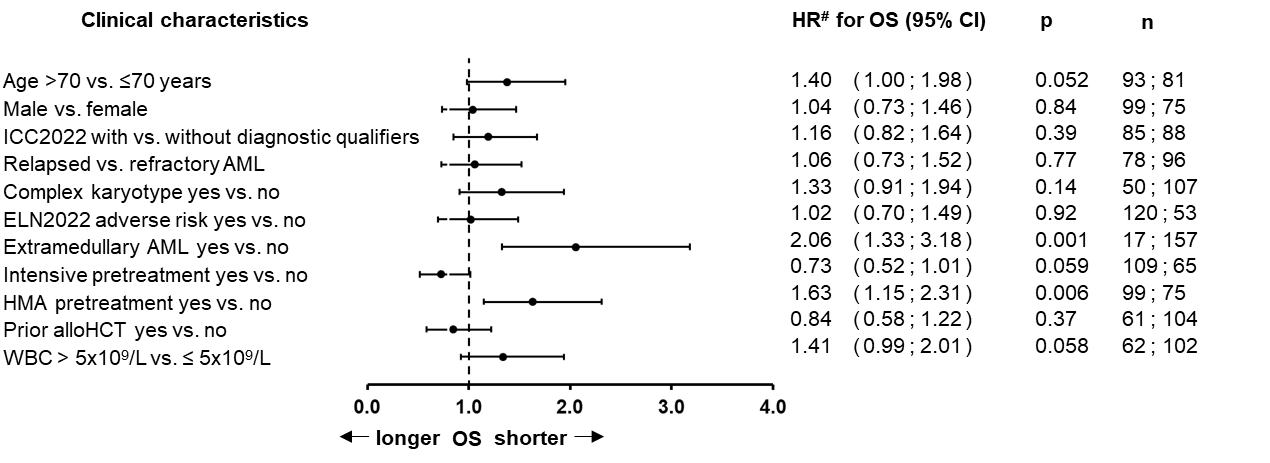


**Supplemental Figure S3:** Forest plot showing univariate analysis of clinical characteristics for OS (molecular cohort n=174). ^#^Hazard ratios greater than or less than 1 indicate an increased or decreased risk, respectively, of an event for the categories listed.


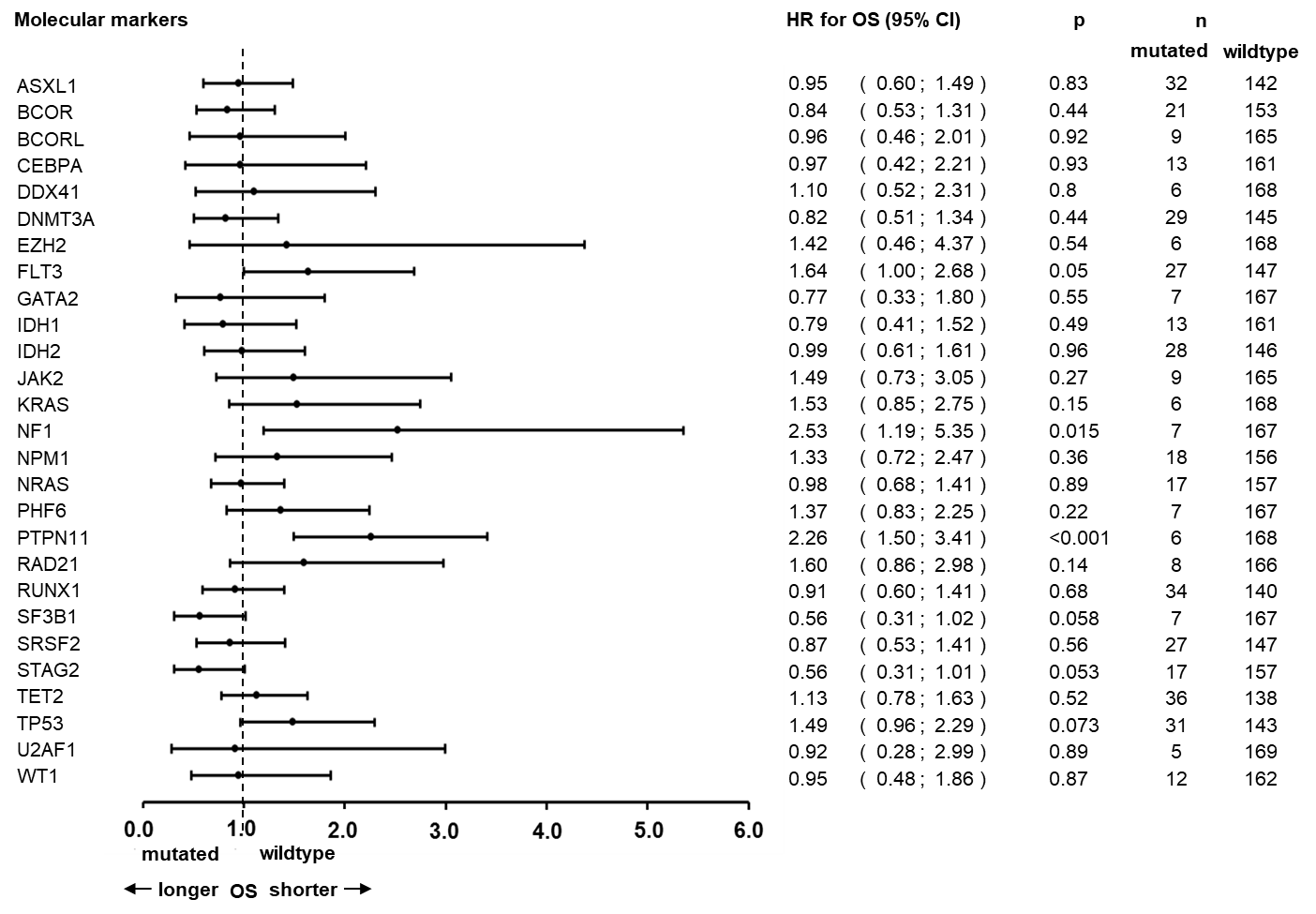


**Supplemental Figure S4:** Forest plot showing univariate analysis of molecular markers for OS (molecular cohort n=174). ^#^Hazard ratios greater than or less than 1 indicate an increased or decreased risk, respectively, of an event for mutated vs wildtype patients.


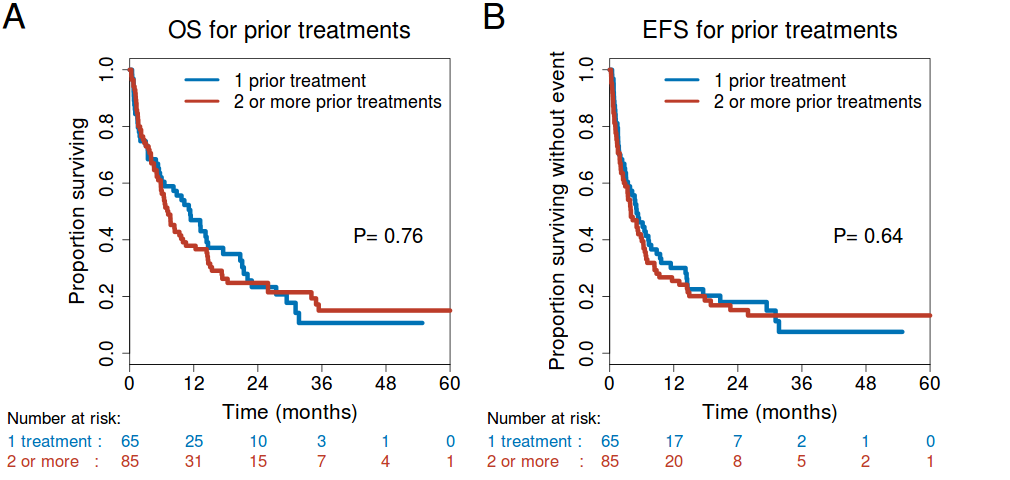


**Supplemental Figure S5:** Kaplan Meier estimates for overall survival stratified by number of previous treatment lines according to the VEN-PRS. (A) Kaplan-Meier estimates for overall survival. (B) Kaplan-Meier estimates for event-free survival.


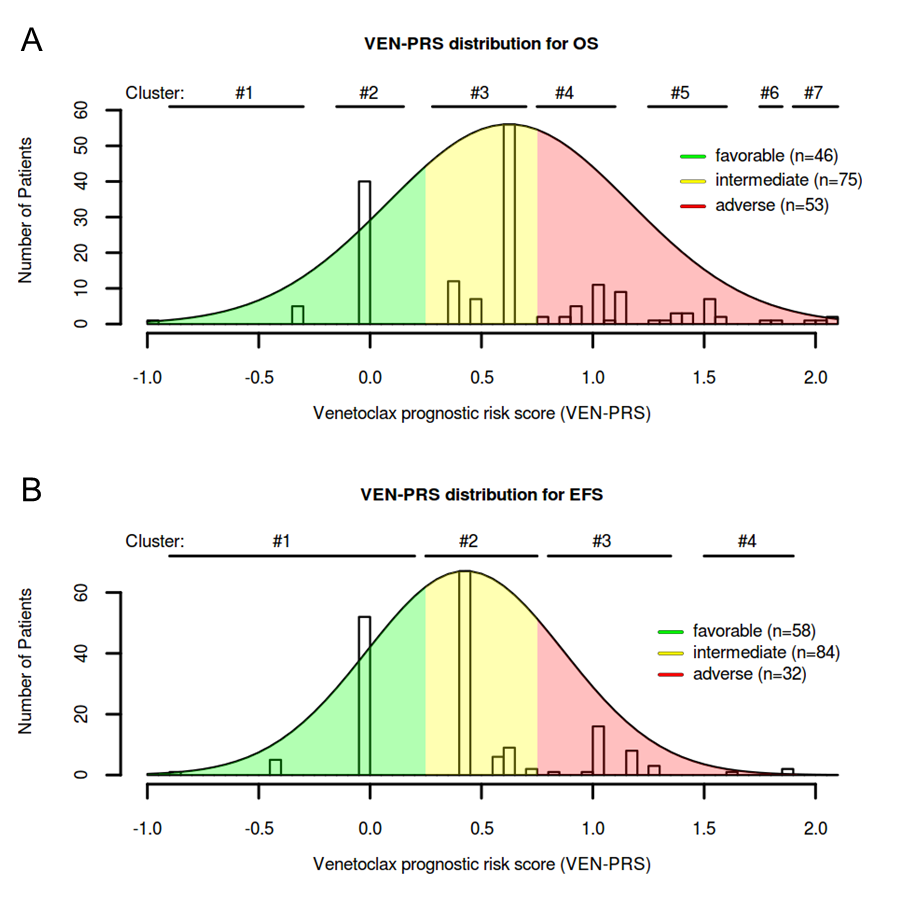


**Supplemental Figure S6:** Distribution of patients according to the individual VEN-PRS (A) for OS, where patients are distributed in 7 clusters, which were combined into favorable, intermediate and adverse risk groups by similar outcomes, and (B) for EFS, where patients are distributed in 4 clusters, which were combined in favorable, intermediate and adverse risk groups by similar outcomes. The selected cutoffs for the risk groups correspond to S_OS/EFS_ <0.25, favorable risk; 0.25≤ S_OS/EFS_ <0.75, intermediate risk; S_OS/EFS_ ≥0.75, adverse risk.


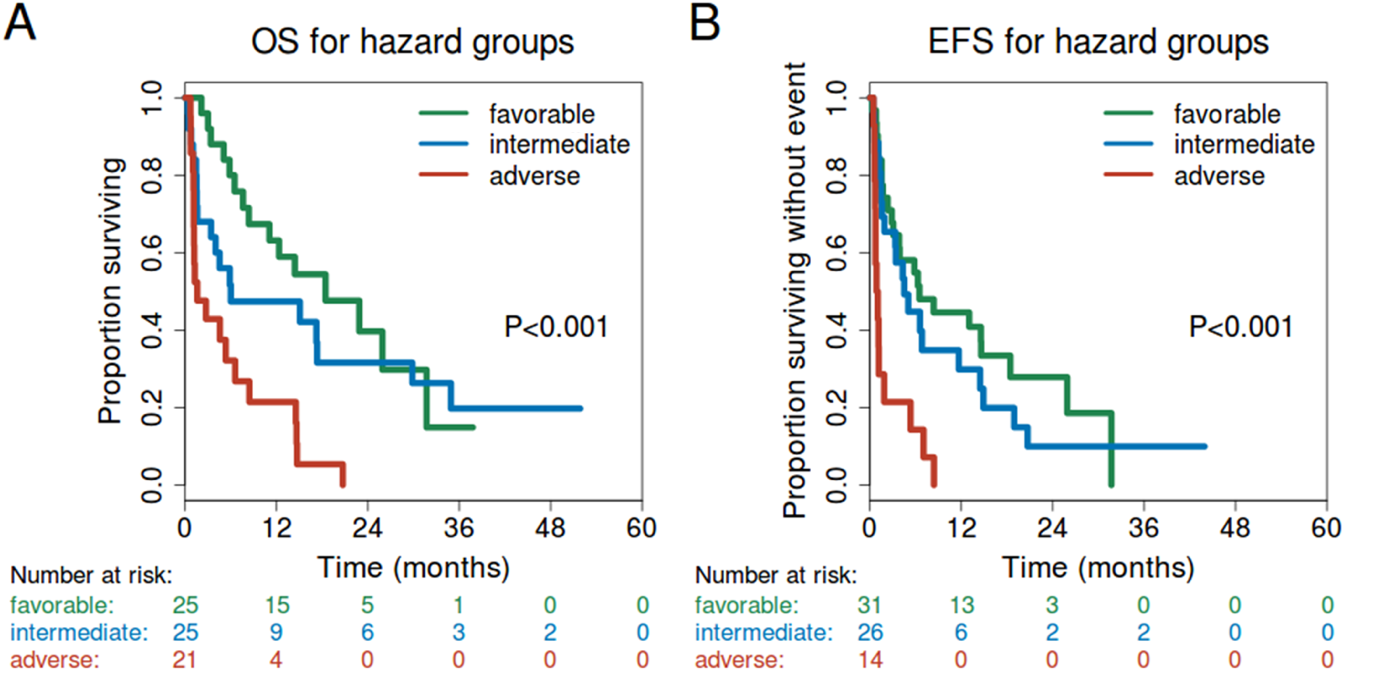


**Supplemental Figure S7:** Kaplan-Meier estimates for survival for relapsed patients according to the VEN-PRS. (A) Kaplan-Meier estimates for overall survival. (B) Kaplan-Meier estimates for event-free survival.


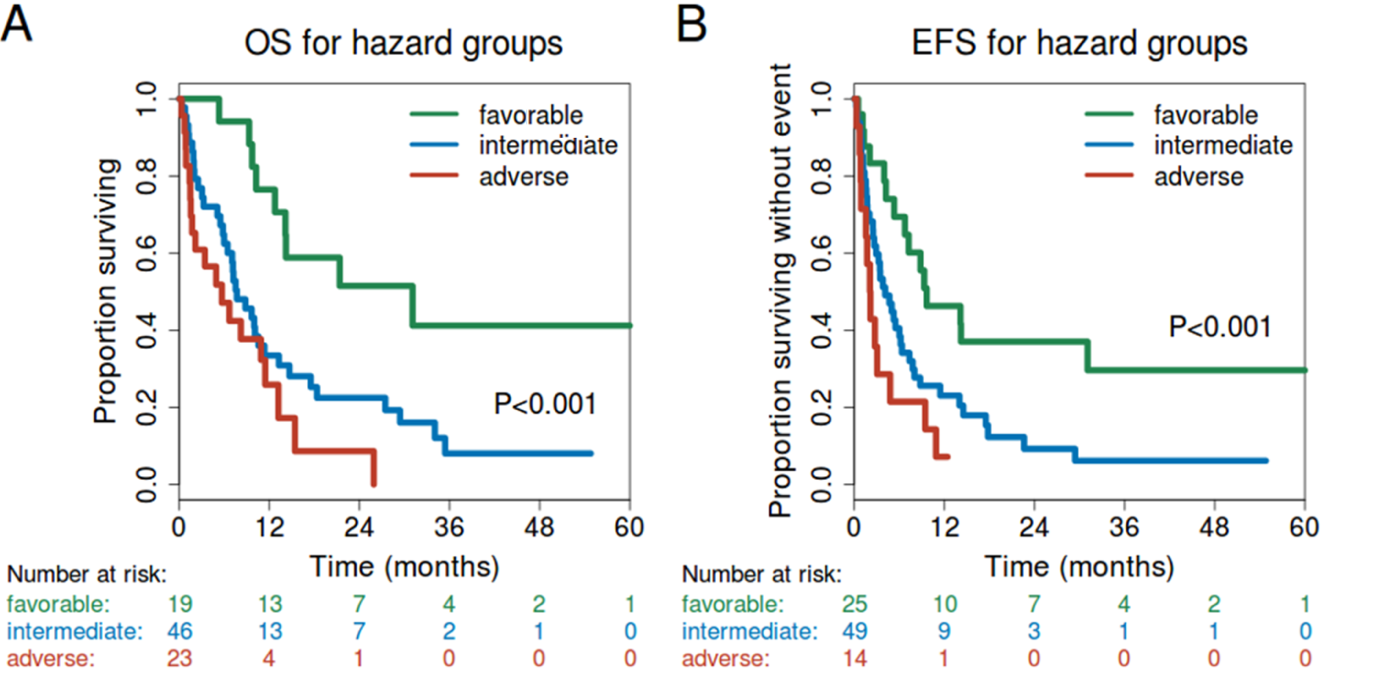


**Supplemental Figure S8:** Kaplan-Meier estimates for survival for refractory patients according to the VEN-PRS. (A) Kaplan-Meier estimates for overall survival. (B) Kaplan-Meier estimates for event-free survival.


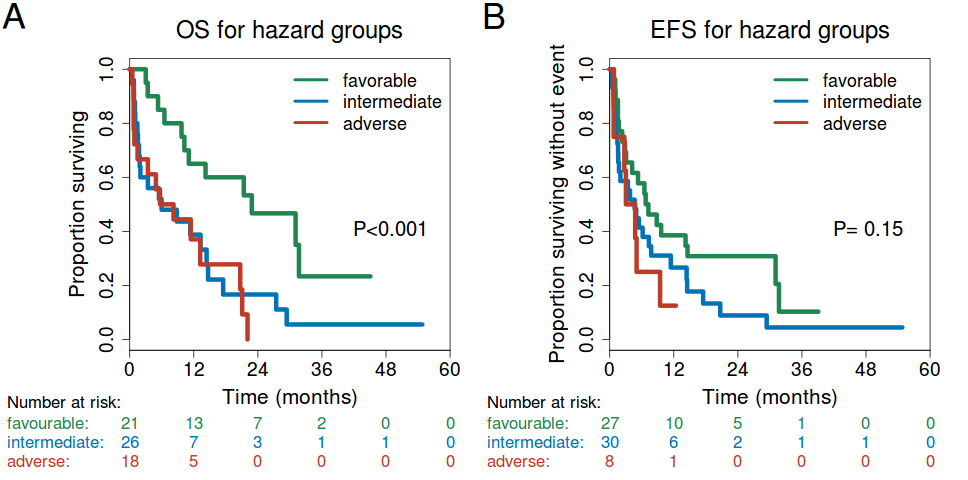


**Supplemental Figure S9:** Kaplan-Meier estimates for survival for patients with one prior line of treatment stratified by VEN-PRS. (A) Kaplan-Meier estimates for overall survival. (B) Kaplan-Meier estimates for event-free survival.


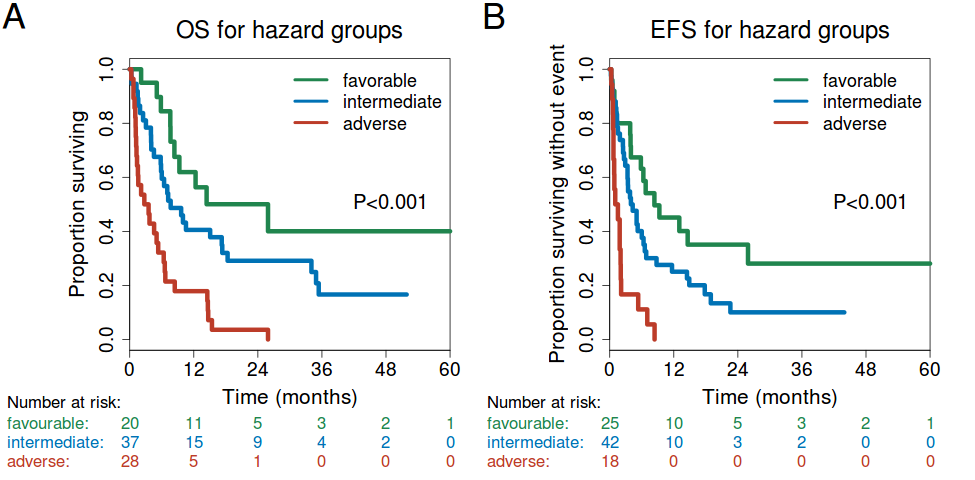


**Supplemental Figure S10:** Kaplan-Meier estimates for survival for patients with two or more prior lines of treatment stratified by VEN-PRS. (A) Kaplan-Meier estimates for overall survival. (B) Kaplan-Meier estimates for event-free survival.


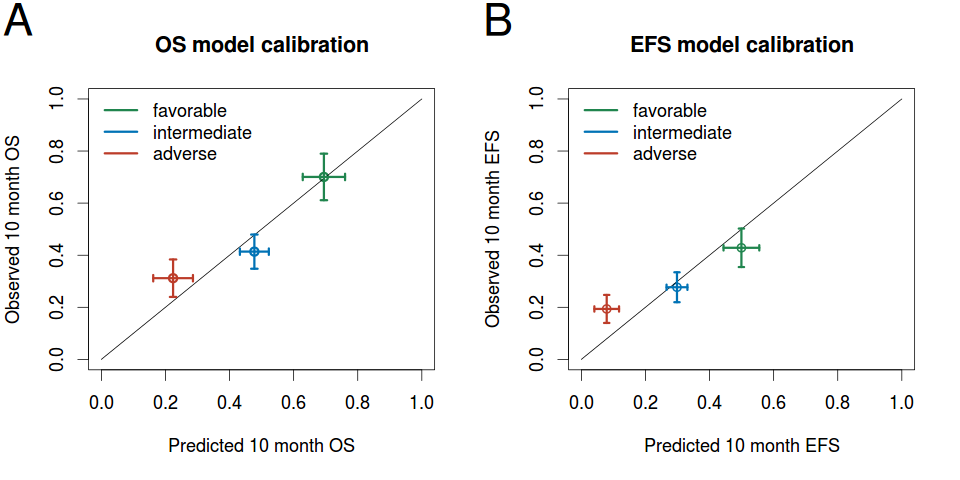


**Supplemental Figure S11:** Calibration analysis of the VEN-PRS shows that the model is well calibrated with three risk groups for OS (A) and EFS (B) at 10 months.


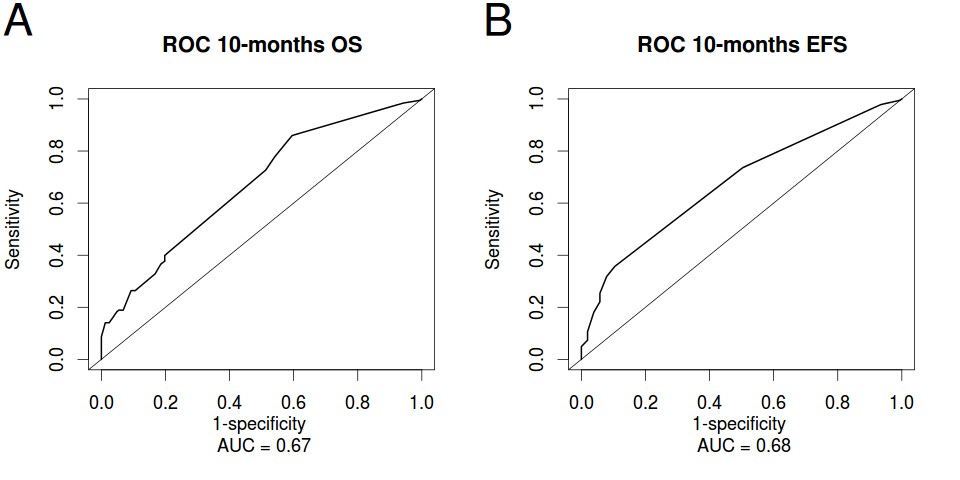
**Supplemental Figure S12**: Discrimination analysis of the VEN-PRS model by calculating receiver operating characteristics (ROC) curves and the area under the curve (AUC) for OS and EFS at 10 months.


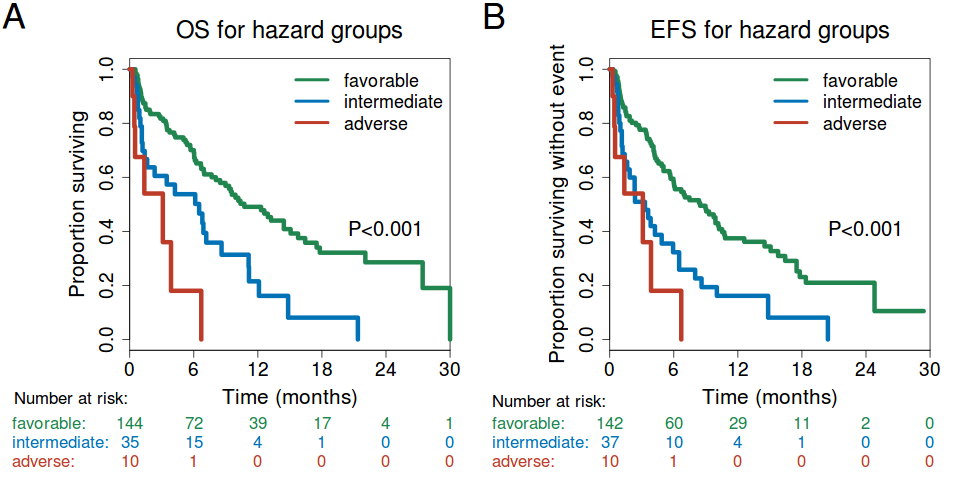


**Supplemental Figure S13:** Kaplan Meier estimates of overall survival (A) and event-free survival (B) according to the VEN-PRS score applied to a cohort of AML patients treated with HMA/VEN in first-line.

*
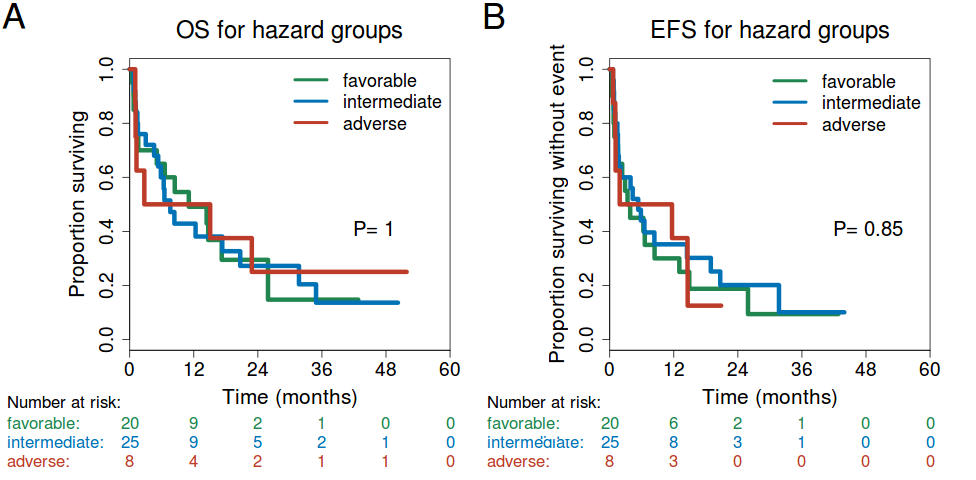
*

**Supplemental Figure S14:** Kaplan Meier estimates of overall survival (A) and event-free survival (B) according to the classification of Breems et al. (7)

*
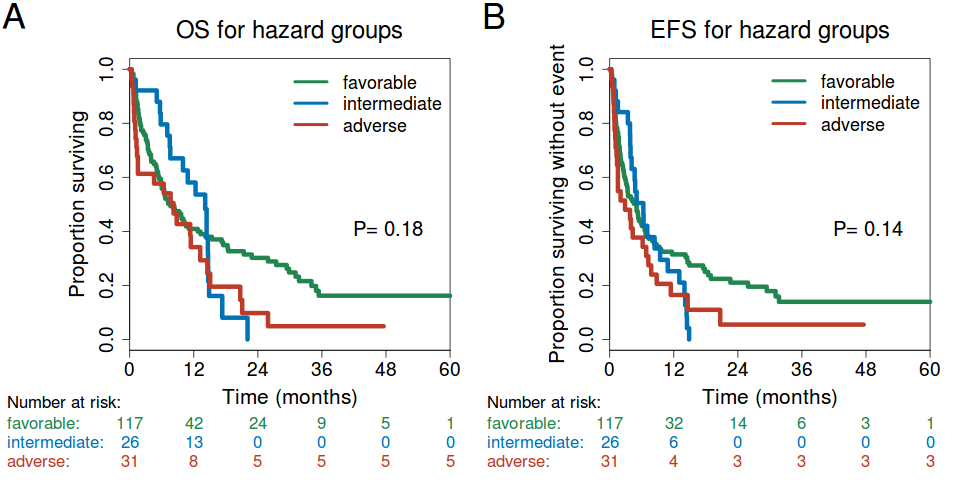
*

**Supplemental Figure S15:** Kaplan Meier estimates of overall survival (A) and event-free survival (B) according to the classification of Doehner et al. (8)

*
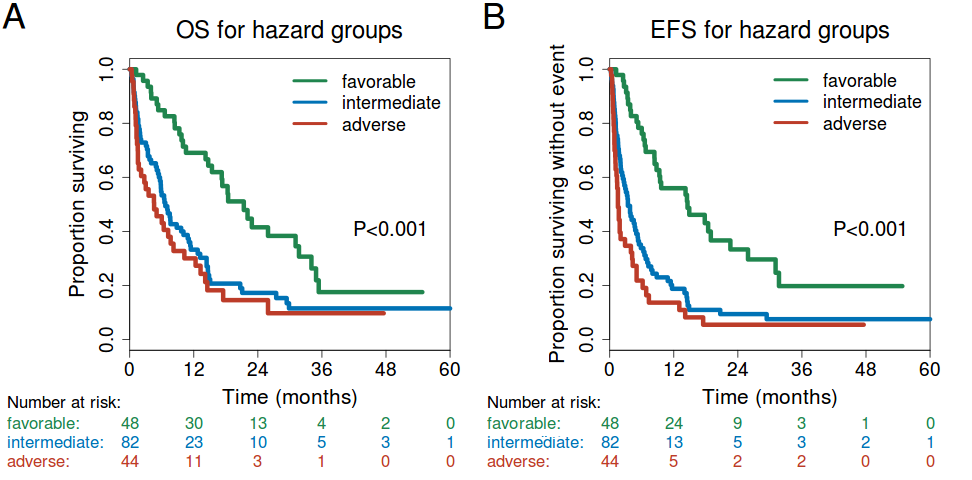
*

**Supplemental Figure S16:** Kaplan Meier estimates of overall survival (A) and event-free survival (B) according to the classification of Gangat et al. (9) (OS: favorable vs. intermediate *p* <0.001; favorable vs. adverse *p* <0.001; intermediate vs. adverse *p*=0.3; EFS: favorable vs. intermediate p <0.001; favorable vs. adverse *p* <0.001; intermediate vs. adverse *p*=0.07).

## **References**

1. Heuser M, Gabdoulline R, Löffeld P, Dobbernack V, Kreimeyer H, Pankratz M, et al. Individual outcome prediction for myelodysplastic syndrome (MDS) and secondary acute myeloid leukemia from MDS after allogeneic hematopoietic cell transplantation. Ann Hematol. 2017;96(8):1361-1372.

2. Döhner H, Wei AH, Appelbaum FR, Craddock C, DiNardo CD, Dombret H, et al. Diagnosis and management of AML in adults: 2022 recommendations from an international expert panel on behalf of the ELN. Blood. 2022;140(12):1345-1377.

3. Schemper M, Smith TL. A note on quantifying follow-up in studies of failure time. Control Clin Trials. 1996;17(4):343-346.

4. Shahswar R, Beutel G, Gabdoulline R, Schwarzer A, Kloos A, Koenecke C, et al. Fludarabine, cytarabine, and idarubicin with or without venetoclax in patients with relapsed/refractory acute myeloid leukemia. Haematologica. 2024;109(1):72-83.

5. Burnham KP, Anderson DR. Multimodel Inference: Understanding AIC and BIC in Model Selection. Sociological methods and research 2004; 33: 261-304.

6. Scrucca L, Fop M, Murphy TB, Raftery AE. mclust 5: Clustering, Classification and Density Estimation Using Gaussian Finite Mixture Models. R J. 2016;8(1):289-317.

7. Breems DA, Van Putten WL, Huijgens PC, Ossenkoppele GJ, Verhoef GE, Verdonck LF, et al. Prognostic index for adult patients with acute myeloid leukemia in first relapse. J Clin Oncol. 2005;23(9):1969-78.

8. Döhner H, Pratz KW, DiNardo CD, Jonas BA, Pullarkat VA, Thirman MJ, et al. ELN Risk Stratification Is Not Predictive of Outcomes for Treatment-Naïve Patients with Acute Myeloid Leukemia Treated with Venetoclax and Azacitidine. Blood. 2022;140(Supplement 1):1441-4.

9. Gangat N, Karrar O, Iftikhar M, McCullough K, Johnson IM, Abdelmagid M, et al. Venetoclax and hypomethylating agent combination therapy in newly diagnosed acute myeloid leukemia: Genotype signatures for response and survival among 301 consecutive patients. Am J Hematol. 2024;99(2):193-202.
